# Integrative Assembly of Heteroleptic Tetrahedra Controlled by Backbone Steric Bulk

**DOI:** 10.1021/jacs.1c01931

**Published:** 2021-04-26

**Authors:** Jacopo Tessarolo, Haeri Lee, Eri Sakuda, Keisuke Umakoshi, Guido H. Clever

**Affiliations:** † Department of Chemistry and Chemical Biology, TU Dortmund University, Otto-Hahn-Straße 6, 44227 Dortmund, Germany; § Department of Chemistry, Hannam University, 1646, Yuseong-daero, Yuseong-gu, Daejeon 34054, Republic of Korea; ‡ Division of Chemistry and Materials Science, Graduate School of Engineering, Nagasaki University, 1-14, Bunkyo-machi, Nagasaki 852-8521, Japan

## Abstract

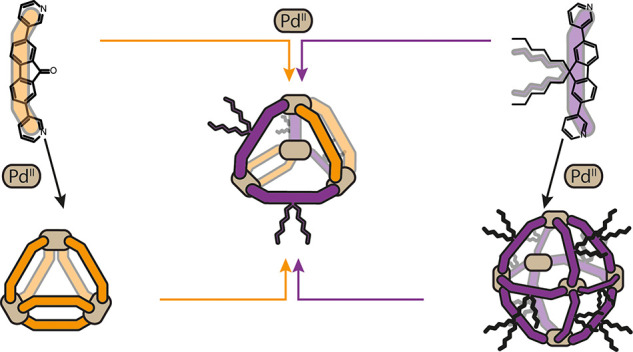

A bent fluorenone-based dipyridyl ligand L^A^ reacts with Pd^II^ cations to a solvent-dependent dynamic library of [Pd_*n*_L_2*n*_] assemblies, constituted by a [Pd_3_L^A^_6_] ring and a [Pd_4_L^A^_8_] tetrahedron as major components, and a [Pd_6_L^A^_12_] octahedron as minor component. Introduction of backbone steric hindrance in ligand L^B^ allows exclusive formation of the [Pd_6_L^B^_12_] octahedron. Combining equimolar amounts of both ligands results in integrative self-sorting to give an unprecedented [Pd_4_L^A^_4_L^B^_4_] heteroleptic tetrahedron. Key to the non-statistical assembly outcome is exploiting the structural peculiarity of the [Pd_4_L_8_] tetrahedral topology, where the four lean ligands occupy two doubly bridged edges and the bulky ligands span the four remaining, singly bridged edges. Hence, the system finds a compromise between the entropic drive to form an assembly smaller than the octahedron and the enthalpic prohibition of pairing two bulky ligands on the same edge of the triangular ring. The emission of luminescent L^A^ is maintained in both homoleptic [Pd_3_L^A^_6_] and heteroleptic [Pd_4_L^A^_4_L^B^_4_].

Coordination-driven self-assembly provides a powerful tool to design and synthesize discrete nanostructured objects with accessible cavities.^1,2^ The resulting metallo-supramolecular assemblies are promising candidates for mimicking functional host systems found in nature, such as enzymes. The dynamic nature of many transition metal–ligand interactions, characterized by precise geometry and directionality, combined with a polytopic ligand structure, allow us to design and self-assemble a plethora of compounds with different shapes, sizes, and properties. Embedded functions, depending on either individual building blocks or their synergistic interaction,^3−7^ may involve host–guest interactions,^8−10^ photoswitching,^11,12^ chirality,^13−15^ chromophore effects,^16−19^ or catalysis,^20−23^ just to name a few.

Besides the formation of single components, dynamic systems consisting of several structures with different topologies may be the result of a self-assembly reaction.^24−29^ So far, most reported metallo-supramolecular compounds carry only one type of ligand, limiting the possibility to exploit applications arising from the implementation of multiple functionalities. To overcome this restriction, we propose to increase structural complexity via the non-statistical integration of a set of different ligands. A first step in this direction is represented by homoleptic assemblies where the same ligand occupies two or more non-identical positions. For example, Lützen reported a [Pd_2_L_4_]@[Pd_4_L_8_] cage-in-ring assembly.^30^ Shionoya differentiated metal positions, thus desymmetrizing a porphyrin ligand.^31^ Our group investigated the controlled formation of [Pd_2_L_3_X_2_] bowls (X = solvent, halides) featuring two different ligand environments.^32,33^ Recently, structural complexity has been increased using non-symmetric ligands.^34−37^ A further approach relies on the structural diversity of [Pd_4_L_8_] assemblies with bis-pyridyl ligands, making it possible to form rings,^38^ interpenetrated double cages,^39,40^ or a tetrahedron-like arrangement, featuring four edges composed of a single ligand and two doubly bridged edges.^5,41−43^

Complexity further increases when chemically different ligands are placed in defined positions, yielding heteroleptic species. To overcome the formation of a statistical mixture,^44^ several strategies have been applied, e.g., exploiting hydrogen-bonding,^45^ templating guests,^46^ shape complementarity,^47−53^ or covalent bridges between ligands.^54^ Herein, we report a system where a bis-monodentate, flat ligand L^A^ self-assembles with Pd^II^ to give a series of [Pd_*n*_L_2*n*_] (*n* = 3, 4, 6) architectures in a solvent-dependent process. Introduction of steric congestion into its backbone gives the bulky ligand L^B^, allowing us to exclusively form a large [Pd_6_L_12_] octahedron. A similar approach was reported by Severin and Hiraoka based on clathrochelate metallo-ligands.^55,56^ We now show that combining lean ligand L^A^ and bulky derivative L^B^ opens a new strategy to form unprecedented [Pd_4_L^A^_4_L^B^_4_] heteroleptic structures. Key to clean, integrative self-sorting is the presence of two non-equivalent edge types in the [Pd_4_L_8_] tetrahedron, combined with control over steric pressure in the ligand backbones.

**Figure 1:**
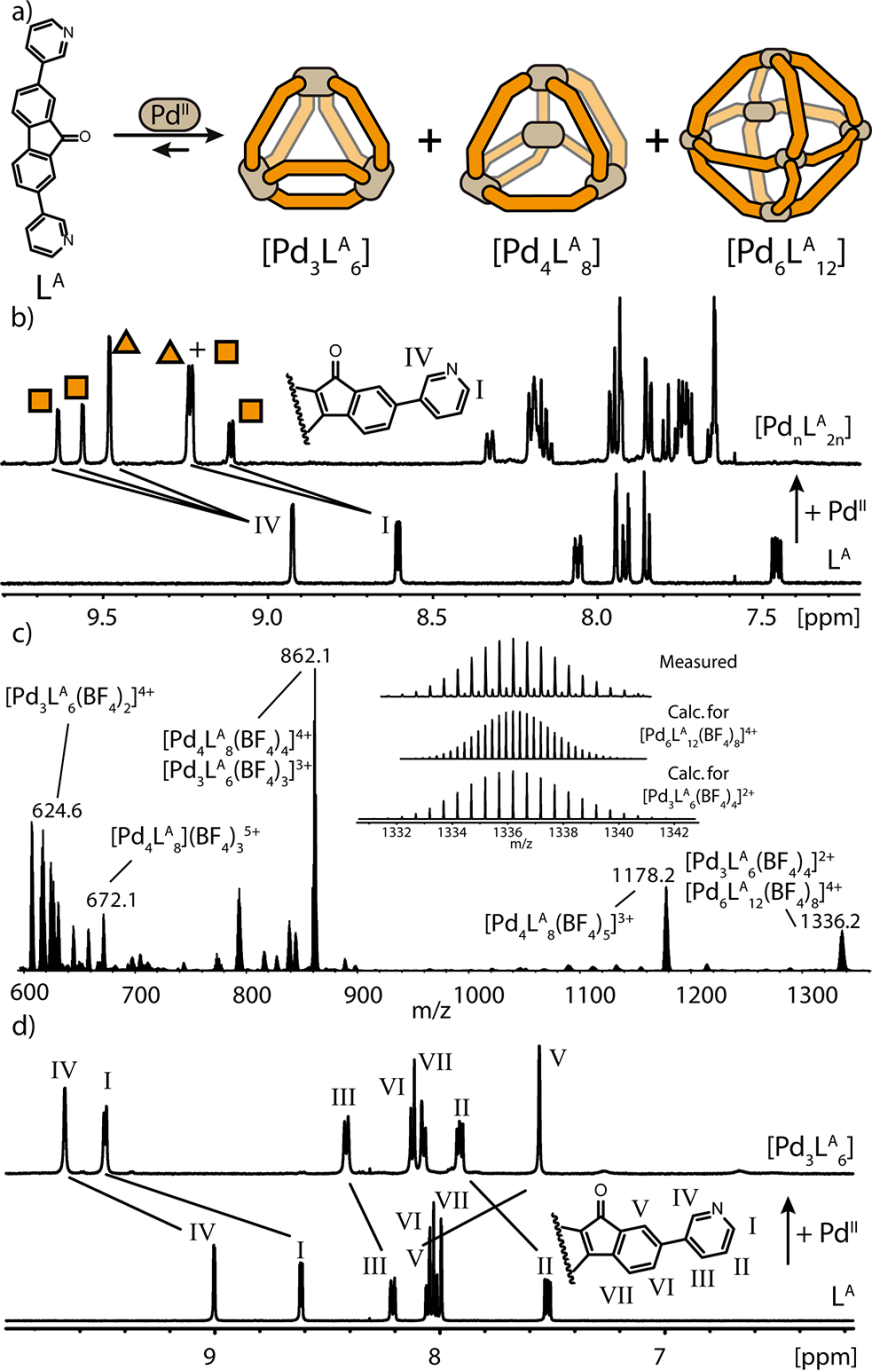
(a) Self-assembly of Pd^II^ and L^A^ forms a [Pd_*n*_L_2*n*_] solvent-dependent library. (b) ^1^H NMR (CD_3_CN, 500 MHz) spectra of [Pd_3_L^A^_6_] (triangles)/[Pd_4_L^A^_8_] (squares) and L^A^. (c) ESI-MS spectrum of [Pd_*n*_L_2*n*_] in CH_3_CN; inset shows the isotopic patterns for [Pd_3_L^A^_6_(BF_4_)_4_]^2+^ and [Pd_6_L^A^_12_(BF_4_)_8_]^4+^. (d) ^1^H NMR (DMSO-*d*_6_, 500 MHz) of [Pd_3_L^A^_6_] and L^A^.

Ligands L^A^ and L^B^ were synthesized by Suzuki cross-coupling starting from 2,7-dibromo-9-fluorenone and 2,7-dibromo-9,9-dihexylfluorene, respectively, with 3-pyridineboronic acid pinacol ester (Supporting Information (SI)). Using 9-substituted fluorene-based backbones makes it possible to obtain non-linear bis-pyridyl ligands, bearing the C=O or alkyl substituents pointing toward one side of the molecule. This generates two binding modes: convex (θ ≈ 90°) with nitrogen donors pointing away from the substituent(s), and concave (θ ≈ 40°) pointing in the same direction (Scheme S4). A similar backbone design was reported to lead to Fe-based helicates and tetrahedra,^57−60^ as well as knots and Borromean rings.^61^

At first, we studied the self-assembly of homoleptic species. Combination of ligand L^A^ with Pd^II^ in a 2:1 ratio led to a solvent-dependent dynamic library of compounds with different nuclearity (Figure 1a). In CD_3_CN, two major components are formed, a [Pd_3_L^A^_6_] triangular ring and a [Pd_4_L^A^_8_] tetrahedron. After coordination to Pd^II^, ^1^H NMR signals are downfield shifted and split into three sets, as clearly observed for proton H_IV_, with a 1:1:2 ratio (Figure 1b). NOESY-NMR allows us to identify two independent sets of signals (SI), while DOSY-NMR shows the presence of two species in solution, with hydrodynamic radii of 11.04 and 12.19 Å, respectively (SI). This is consistent with the formation of a [Pd_3_L^A^_6_] ring and a [Pd_4_L^A^_8_] tetrahedron, the latter generating two set of ^1^H NMR signals due to two non-equivalent ligand positions. Support comes from high-resolution ESI-MS analysis, showing a series of signals for [Pd_3_L^A^_6_(BF_4_)_*n*_]^*m*+^ (*n* = 2–4; *m* = 4–2) and [Pd_4_L^A^_8_(BF_4_)_*n*_]^*m*+^ (*n* = 4, 5; *m* = 4, 3) (Figure 1c). Interestingly, the signal at *m*/*z* = 1336.2 reveals the presence of [Pd_3_L^A^_6_(BF_4_)_4_]^2+^ as major and higher-nuclear [Pd_6_L^A^_12_(BF_4_)_8_]^4+^ as minor components (Figure 1c, inset). Self-assembly in DMSO-*d*_6_ results in only [Pd_3_L^A^_6_] ring formation, as confirmed by ^1^H NMR (Figure 1d), DOSY-NMR (*r*_H_ = 13.20 Å), and ESI-MS analysis (SI). The structures for all three [Pd_*n*_L_2*n*_] components have been determined by single-crystal X-ray diffraction (SCXRD) analysis (Figure 2). Needle-shaped crystals of the trimetallic [Pd_3_L^A^_6_] were obtained by vapor diffusion of toluene into a DMSO solution. The compound has the expected triangular geometry, where Pd^II^ metal centers occupy the vertices while pairs of ligands sit on the edges (Figure 2a). The carbonyl backbone substituent adopts two positions, one pointing outside the ring cavity while the other points toward the π-surface of the neighboring ligand. The distance between the fluorenone oxygen and the 5-membered ring centroid of L^A^ is 3.07 Å, suggesting the incompatibility of a bulkier backbone substituent with this structure. Diffusion of ethyl acetate into a DMF solution yielded single crystals of [Pd_4_L^A^_8_], yielding a tetrahedral structure with Pd^II^ centers on the vertices and ligands bridging the edges (Figure 2b). In this case, L^A^ occupies two non-equivalent positions: four edges (“edge 1”) are composed of one ligand, while the two remaining edges (“edge 2”) accommodate a pair of ligands. In edge 1, the carbonyl substituent points outside the tetrahedral cavity, with L^A^ adopting a convex binding mode (θ ≈ 80°). In edge 2, the carbonyl group of one L^A^ points toward the π-surface of its neighbor (C=O−π-C_5_ centroid = 3.53 Å), while the other carbonyl group points inside the cavity, featuring a concave binding mode (θ ≈ 40°). Finally, diffusion of 1,4-dioxane into the [Pd_*n*_L^A^_2*n*_] (*n* = 3, 4, 6) CH_3_CN solution resulted in single crystals of [Pd_6_L^A^_12_], suitable for synchrotron diffraction analysis. The compound crystallizes in the *R*3̅ space group as a pair of enantiomeric [Pd_6_L^A^_12_] octahedra (Figure 2c). Pd^II^ cations occupy the vertices, while the edges feature one ligand L^A^ each, with all carbonyl groups pointing outside the cavity. Comparing the three [Pd_*n*_L^A^_2*n*_] structures suggests that only the octahedron, the sole structure without C=O−π interactions, should be able to accommodate sterically demanding ligands on all edges.

**Figure 2:**
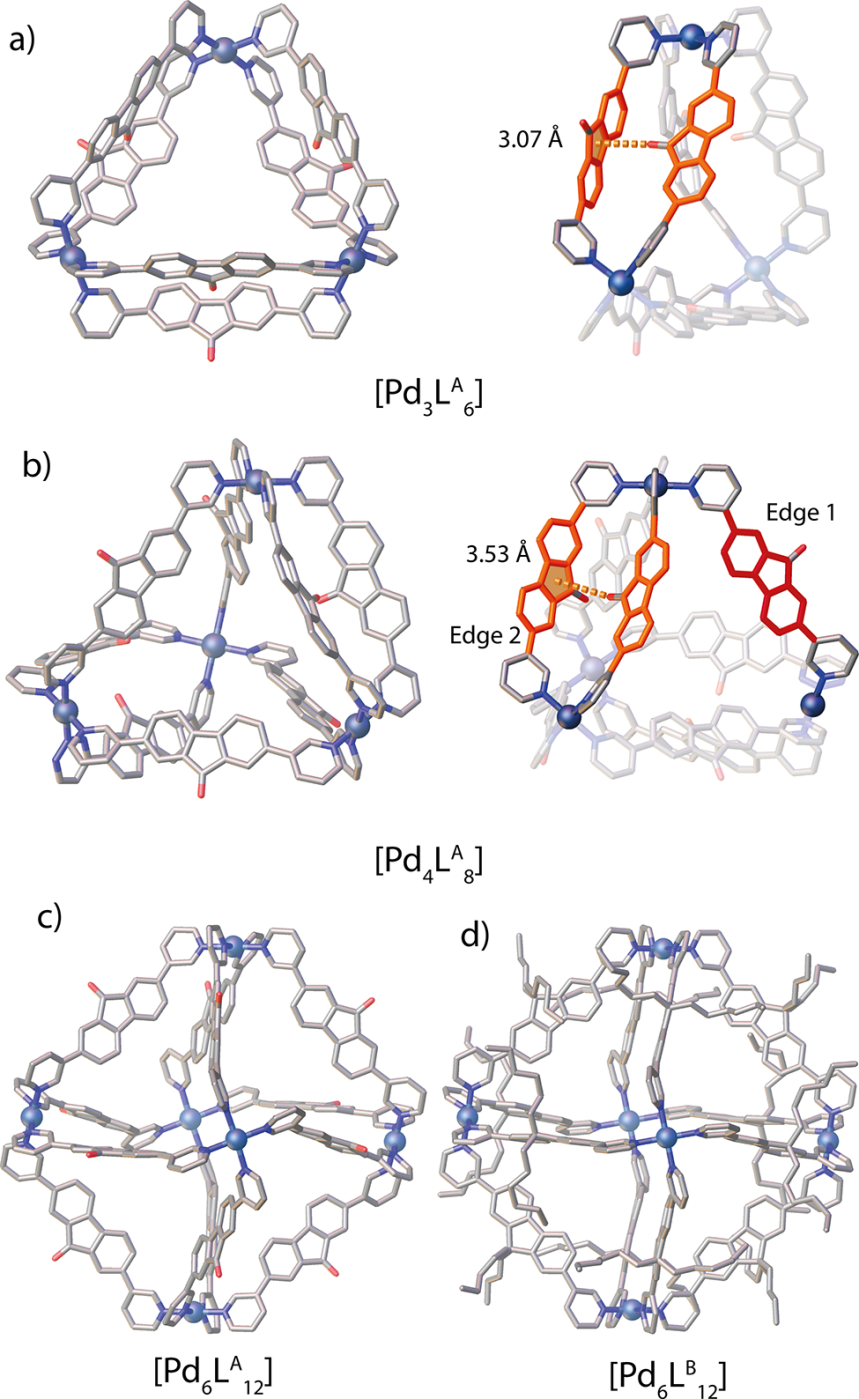
SCXRD structures of (a) [Pd_3_L^A^_6_] ring (left), highlighting the C=O−π interaction (right); (b) [Pd_4_L^A^_8_] tetrahedron (left), highlighting edge 1 (red), edge 2 (orange), and the C=O−π interaction (right); (c) [Pd_6_L^A^_12_] octahedron (one enantiomer shown); and (d) [Pd_6_L^B^_12_] octahedron. Counterions, solvent molecules, hydrogen atoms, and disorder are omitted for clarity. Color code: Pd, metallic blue; N, blue; O, red; C, gray.

To explore this possibility, L^B^ was synthesized by replacing the 9-fluorenone with a 9,9-dihexylfluorene backbone. Self-assembly of L^B^ with Pd^II^ cations in a 2:1 stoichiometry led to the formation of a single species, identified as a [Pd_6_L^B^_12_] octahedron (Figure 3a). Upon complexation of Pd^II^, the ^1^H NMR signals of L^B^ are downfield shifted and slightly broadened (Figure 3b). DOSY analysis confirmed a single species in CD_3_CN (*r*_H_ = 15.22 Å) and DMSO-*d*_6_ (*r*_H_ = 18.66 Å, SI), while pointing to differences in the solvent shell dynamics around the heavily alkyl-decorated [Pd_6_L^B^_12_] species. The high nuclearity was confirmed by HR-ESI-MS, where a series of peaks for [Pd_6_L^B^_12_(BF_4_)_*n*_]^*m*+^ (*n* = 3–6; *m* = 9–6) were identified (Figure 3c). Moreover, single crystals were obtained from vapor diffusion of toluene into a DMSO solution. The compound crystallizes as an octahedron with a structure analogous to that of [Pd_6_L^A^_12_]. L^B^ sits on the edges and coordinates in the convex mode, and all hexyl chains point outside the cavity (Figure 2d). From these results we inferred that the steric bulk in the backbones of L^B^ prevents two ligands from being direct neighbors on the same edge, thus averting formation of entropically favored homoleptic species [Pd_3_L_6_] or [Pd_4_L_8_].

**Figure 3:**
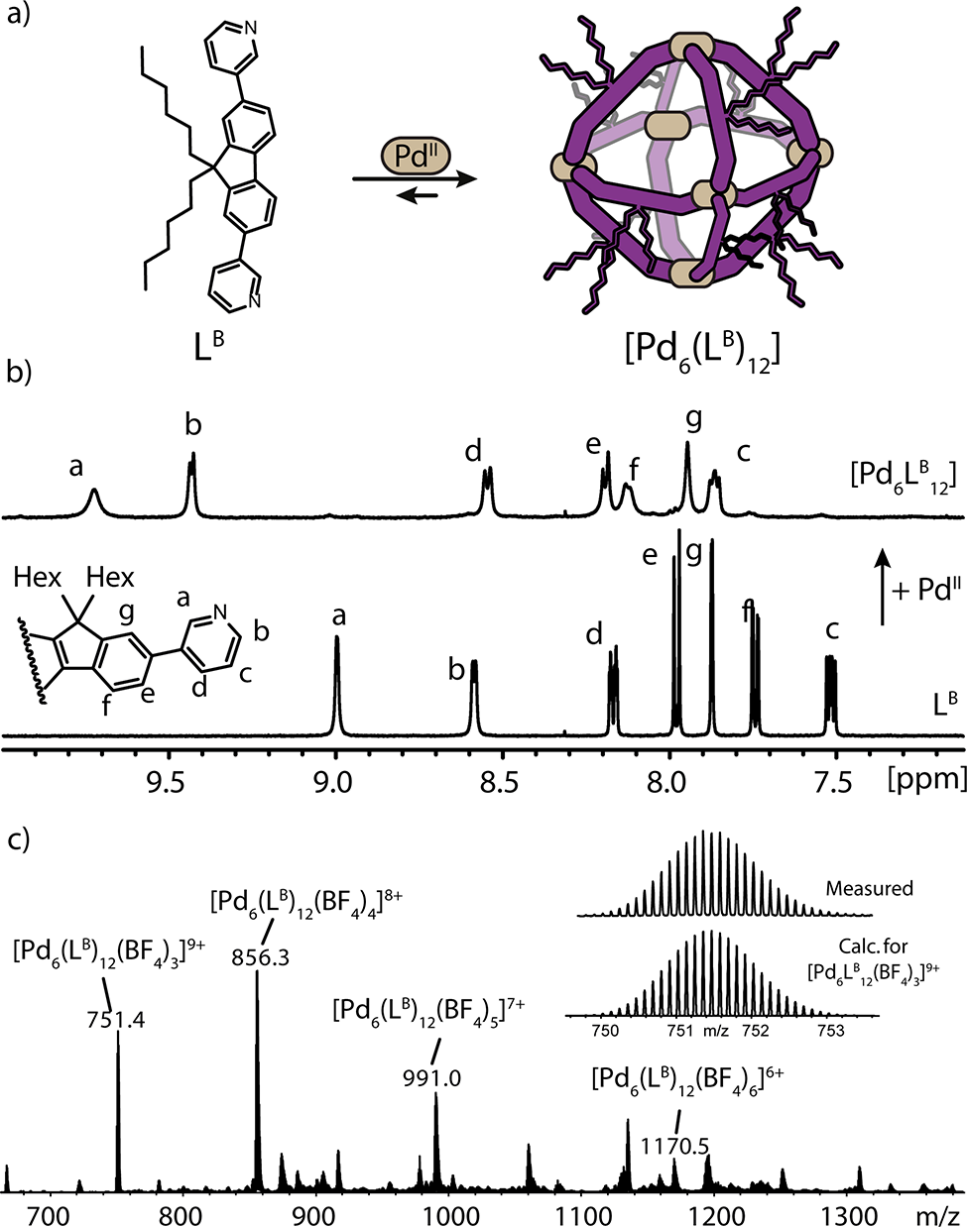
(a) Self-assembly of Pd^II^ and L^B^ to form [Pd_6_L^B^_12_]. (b) ^1^H NMR (DMSO-*d*_6_, 500 MHz) spectra of [Pd_6_L^B^_12_] and L^B^. (c) ESI-MS spectrum of [Pd_6_L^B^_12_] with isotopic pattern of [Pd_6_L^B^_12_(BF_4_)_3_]^9+^ shown in the inset.

Looking at the [Pd_4_L^A^_8_] tetrahedral structure (Figure 2b) reveals that while edge 2 must fit two ligands, edge 1 can accommodate a single ligand with a bulkier backbone, adopting a convex binding mode with substituents pointing outside the cavity. Based on this assumption, we postulated the formation of an unprecedented heteroleptic [Pd_4_L^A^_4_L^B^_4_] tetrahedron, with four L^A^ sitting on edges 1, while ligands L^B^ occupy edges 2. Hence, Pd^II^, L^A^, and L^B^ were mixed in a 1:1:1 ratio in DMSO-*d*_6_ at 70 °C for 1 h, indeed resulting in the exclusive formation of a [Pd_4_L^A^_4_L^B^_4_] heteroleptic tetrahedron (Figure 4b). ^1^H NMR signals show downfield shifting, without signs of any homoleptic assemblies (Figure 4c). The presence of both L^A^ and L^B^ within the same structure is supported by NOESY-NMR, showing a number of cross-signals, e.g., between H_b_ and H_I_ (SI). DOSY-NMR clearly shows a single species (*r*_H_ = 15.11 Å), bigger than [Pd_3_L^A^_6_], but smaller compared to [Pd_6_L^B^_12_] in the same solvent (Figure S36). Furthermore, in the ESI-MS spectrum, a series of peaks for [Pd_4_L^A^_4_L^B^_4_(BF_4_)_*n*_]^*m*+^ (*n* = 1–5; *m* = 7–3) were identified (Figure 4d). Due to the dynamic nature of the metallo-supramolecular system, the same result was obtained in the fashion of a “cage-to-cage transformation” when two equimolar solutions of [Pd_3_L^A^_6_] and [Pd_6_L^B^_12_] were mixed (Figure S33). Despite a longer reaction time (Figure S34) than starting from free ligands plus Pd^II^, in agreement with our previous findings,^49^ this proves that [Pd_4_L^A^_4_L^B^_4_] is a thermodynamic product.

**Figure 4:**
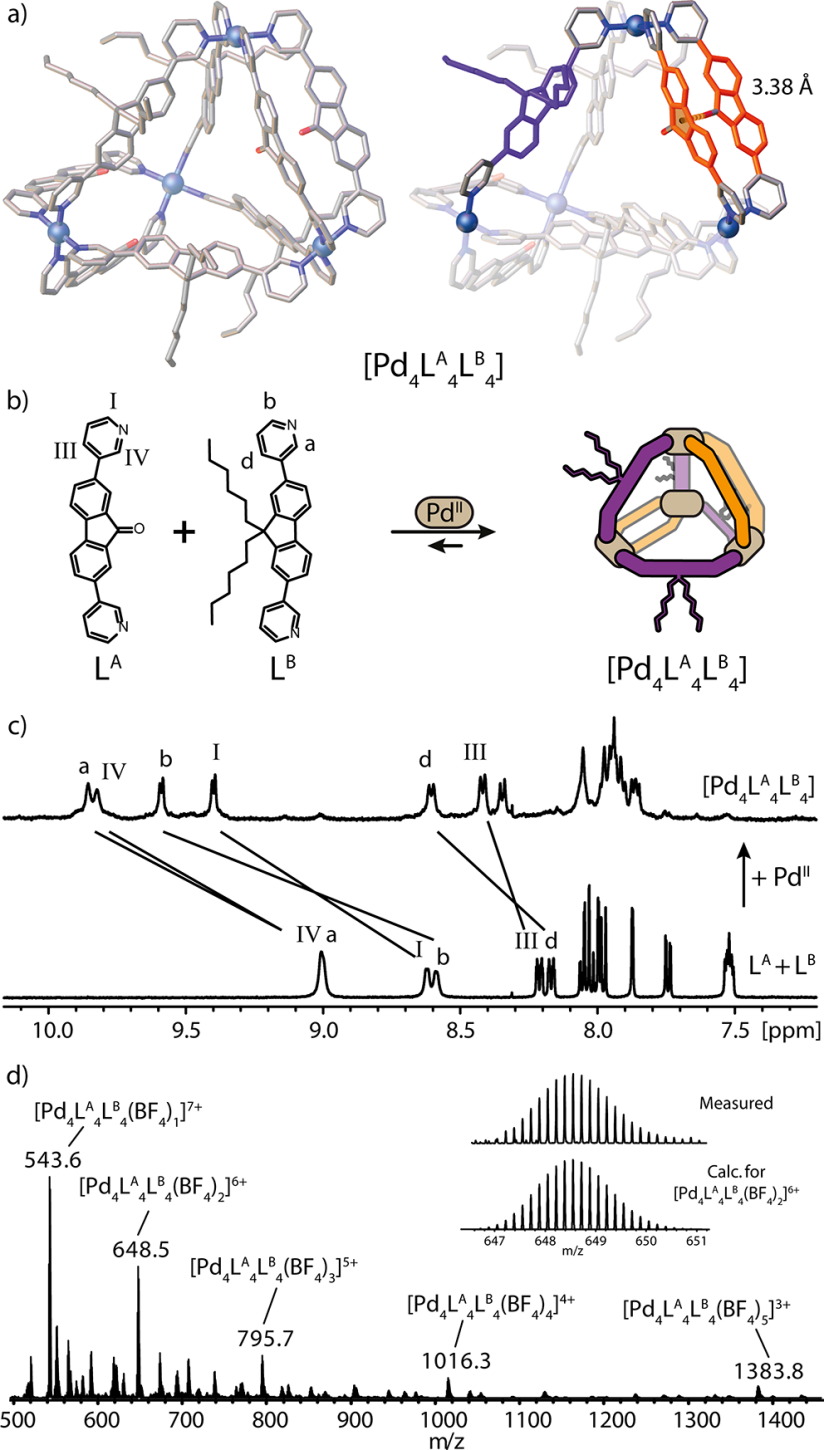
(a) SCXRD structure of [Pd_4_L^A^_4_L^B^_4_] (left), highlighting L^A^ (orange) and L^B^ (purple) positions and the C=O−π interaction (right). Counterions, solvent molecules, hydrogen atoms, and disorder are omitted for clarity. Color code: Pd, metallic blue; N, blue; O, red; C, gray. (b) Self-assembly of Pd^II^ with L^A^ and L^B^ forms [Pd_4_L^A^_4_L^B^_4_]. (c) ^1^H NMR (DMSO-*d*_6_, 500 MHz) spectra of [Pd_4_L^A^_4_L^B^_4_] and a 1:1 mixture of L^A^ and L^B^. (d) ESI-MS spectrum of [Pd_4_L^A^_4_L^B^_4_], with isotopic pattern of [Pd_4_L^A^_4_L^B^_4_(BF_4_)_3_]^6+^ shown in the inset.

Structural analysis of single crystals, from benzene vapor diffusion into DMSO, ultimately proved the formation of a [Pd_4_L^A^_4_L^B^_4_] heteroleptic tetrahedron (Figure 4a). To the best of our knowledge, this is the first example of such a [M_4_L^A^_4_L^B^_4_] heteroleptic assembly topology. As postulated, four ligands L^A^ are accommodated on edges 2, with the C=O group either pointing inside the cavity or facing the π-surface of neighboring L^A^ (C=O−π-C_5_ centroid = 3.38 Å, Figure 4a). In addition, four ligands L^B^ are sitting on edges 1, adopting a convex binding mode with hexyl chains pointing outside the cavity (Figure 4a, purple backbone).

Next, we investigated guest binding of one aliphatic and two aromatic bis-sulfonates (Scheme S5) with [Pd_3_L^A^_6_], [Pd_6_L^B^_12_] and [Pd_4_L^A^_4_L^B^_4_] in DMSO-*d*_6_. In all cases, ^1^H NMR titrations show interaction of the guests with the cage’s inner cavity, indicated by a shift of inward-pointing protons (SI). Signal broadening and onset of precipitation prevented us from determining association constants. ESI-MS analysis, however, suggests that the maximal number of hosted guests is controlled by the assembly size. While for [Pd_3_L^A^_6_] we only observed interaction with one guest, for tetrahedra [Pd_4_L^A^_8_] and [Pd_4_L^A^_4_L^B^_4_] binding of two guests was detected, and large octahedron [Pd_6_L^B^_12_] was even found to bind up to three guest molecules (SI).

Finally, we investigated the photophysical properties of the systems (Figure 5). In DMSO, L^A^ shows an emission band centered at 555 nm that is blue-shifted to 532 nm upon Pd^II^ complexation in either homoleptic [Pd_3_L^A^_6_] or heteroleptic [Pd_4_L^A^_4_L^B^_4_]. While this indicates that the emissive properties of ligand L^A^ are retained in the heteroleptic tetrahedron, our platform allows to introduce additional functionality through modification of L^B^. It is worth noting that in Pd-mediated assemblies luminescence quenching is frequently observed,^15,39^ and only few examples of emissive cages have been reported so far.^18,62−65^

**Figure 5:**
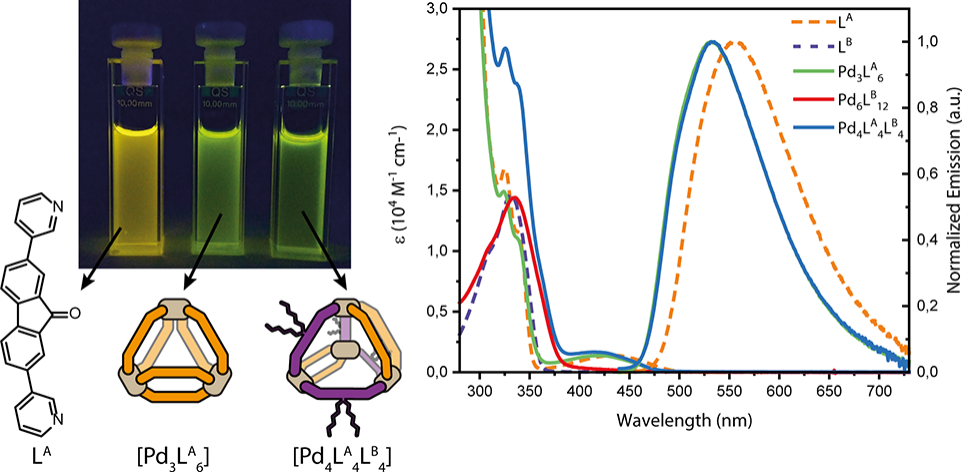
Absorption spectra of ligands and cages (5.0 × 10^–4^ M referred to ligand concentration; DMSO) and normalized emission spectra of L^A^, [Pd_3_L^A^_6_], and [Pd_4_L^A^_4_L^B^_4_] (1.4 × 10^–4^ M; λ_ex_ = 430 nm).

To conclude, we report a new strategy for the non-statistical, integrative self-assembly of a previously unreported [M_4_L^A^_4_L^B^_4_] heteroleptic cage topology. Key factors are the use of bis-monodentate ligands, able to adopt a concave or convex binding mode, the precise introduction of backbone steric hindrance, and a balance between the entropic tendency to form small assemblies and the enthalpic disadvantage to pair bulky substituents on a single edge. The preservation of ligand emission properties in the Pd-mediated assemblies opens potential toward application as multifunctional devices and materials in fields such chiroptical sensing, donor–acceptor systems, and photoredox catalysis.

## Supporting Information Available

Synthetic procedures, NMR, MS, SCXRD, and spectroscopic data, including Schemes S1–S5, Figures S1–S48, Tables S1–S3 (PDF)

## Accession Codes

CCDC 2061340, 2061344, 2061345, 2061347, 2061350, and 2061351 contain the supplementary crystallographic data for this paper. These data can be obtained free of charge via www.ccdc.cam.ac.uk/data_request/cif, or by emailing data_request@ccdc.cam.ac.uk, or by contacting The Cambridge Crystallographic Data Centre, 12 Union Road, Cambridge CB2 1EZ, UK; fax: +44 1223 336033.
