# Supplementary material for: Integrative Assembly of Heteroleptic Tetrahedra Controlled by Backbone Steric Bulk
Source: J Am Chem Soc. 2021 Apr 26;143(17):6339–44. doi: 10.1021/jacs.1c01931 (PMC8154538; doi:10.1021/jacs.1c01931)
Supplement: Supplementary file 1 — ja1c01931_si_001.pdf [file ja1c01931_si_001.pdf]

Integrative Assembly of Heteroleptic Tetrahedra Controlled by Backbone Steric Bulk

Jacopo Tessarolo,[†] Haeri Lee,[§] Eri Sakuda,[†,‡] Keisuke Umakoshi,[‡] Guido H. Clever[†,*]

[†]Faculty of Chemistry and Chemical Biology, TU Dortmund University, Otto-Hahn-Straße 6, 44227 Dortmund, Germany.
[§]Department of Chemistry, Hannam University, 1646, Yuseong-daero, Yuseong-gu, Daejeon, 34054, Republic of Korea.
[‡]Division of Chemistry and Materials Science, Graduate School of Engineering, Nagasaki University, 1-14, Bunkyo-machi, Nagasaki 852-8521, Japan.

Supporting Information

1. General procedures 2
2. Synthesis 3
  2.1 Ligand synthesis 3
    2.1.1 Synthesis of L^A. 3
    2.1.2 Synthesis of L^B. 5
  2.2 Cage synthesis 8
    2.2.1 Synthesis of $[Pd_nL^A_{2n}]$ in CD_3CN. 8
    2.2.2 Synthesis of $[Pd_3L^A_6]$ in DMSO-d_6. 11
    2.2.3 Synthesis of $[Pd_6L^B_{12}]$. 14
    2.2.4 Synthesis of $[Pd_4L^A_4L^B_4]$. 20
    2.2.5 Cage to Cage transformation. 23
  2.3 DOSY NMR comparison 24
3. Ligand binding modes 26
4. Single crystal X-ray diffraction 27
  4.1. Data collection details of $[Pd_3L^A_6]$ and $[Pd_6L^A_{12}]$ 27
    4.1.1. Refinement details for $[Pd_3L^A_6]$ and $[Pd_6L^A_{12}]$ 28
  4.2. Data collection details of $[Pd_4L^A_8]$, $[Pd_6L^B_{12}]$, $[Pd_4L^A_4L^B_4]$ and L^B 28
    4.2.1. Refinement details for $[Pd_4L^A_8]$, $[Pd_6L^B_{12}]$, $[Pd_4L^A_4L^B_4]$ and L^B 28
5. Photophysical studies 31
6. Host-Guest studies 32

6.1. Host-guest studies of [$Pd_3L^A{}_6$]. 33
6.2. Host-guest studies of [$Pd_6L^B{}_{12}$]. 36
6.3. Host-guest studies of [$Pd_4L^A{}_4L^B{}_4$]. 39
7. References 42

1. General procedures

All chemicals, except otherwise specified, were obtained from commercial sources and used without further purification. Recycling gel permeation chromatography was performed on a JAI LC-9210 II NEXT GPC system or a JAI LaboAce LC-5610 system using chloroform as eluent (HPLC grade). NMR spectroscopic data was measured on the spectrometers Bruker AV 500 Avance NEO and AV 600 Avance III HD. For ^{1}H and ^{13}C NMR spectra, chemical shifts were calibrated to the solvent lock signal. Chemical shifts δ are given in ppm, coupling constants J in Hz. The following abbreviations are used to describe signal multiplicity for 1H NMR spectra: s: singlet, d: doublet, t: triplet, dd: doublet of doublets; dt: doublet of triplets; m: multiplet, br: broad. All signals were assigned with the aid of 2D NMR spectra. All spectra were recorded in standard 5 mm NMR tubes at room temperature. ^{1}H DOSY NMR spectra were recorded with a dstebpgp3s pulse sequence with diffusion delays D20 of 0.09-0.10 s and gradient powers P30 of 1200 to 1400 µs in CD_3CN and diffusion delays D20 of 0.10-0.12 s and gradient powers P30 of 2800 to 3100 µs in DMSO-d_6. Mass spectrometry data were measured on Bruker ESI-timsTOF and Bruker compact high-resolution LC mass spectrometers (positive/negative mode). For calibration of the TIMS and TOF devices, Agilent ESI-Low Concentration Tuning Mix was used.

2. Synthesis

2.1 Ligand synthesis

2.1.1 Synthesis of L^A.

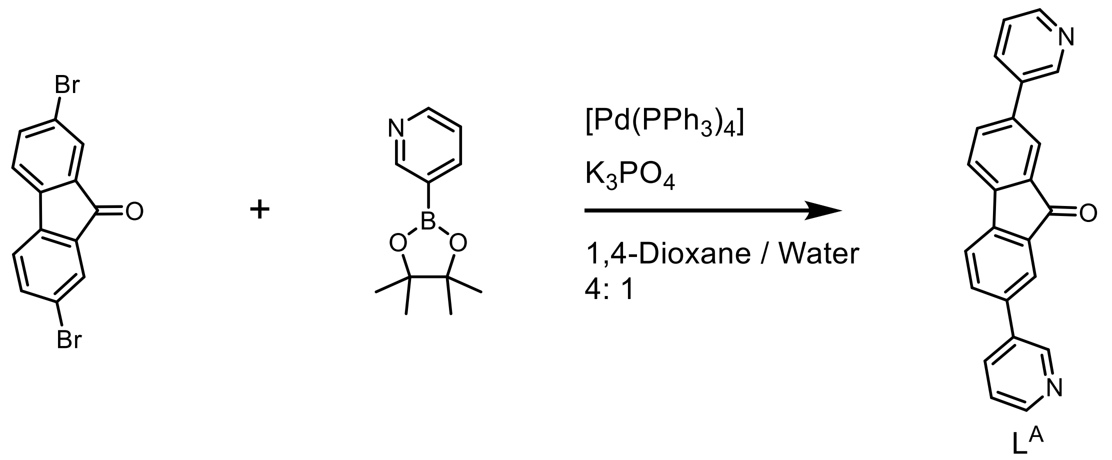

Scheme S1. Synthesis of L^A

A 5 mL aqueous solution of K_3PO_4 (380 mg, 1.8 mmol) was added to a 1,4-dioxane (20 mL) solution of 2,7-dibromo-9-fluorenone (300 mg, 0.9 mmol) and pyridine-3-boronic acid pinacol ester (450 mg, 2.2 mmol). The resulting solution was thoroughly degassed and $[Pd(PPh_3)_4]$ (82 mg, 0.07 mmol) were added under nitrogen atmosphere. After stirring at 90 °C overnight, the mixture was allowed to cool down to room temperature and 30 mL of dichloromethane were added. The solution was washed with brine (3 x 15 mL) and dried over $MgSO_4$. The solvent was removed under vacuum affording an orange powder. The crude product was purified through column chromatography (silica) using ethyl acetate as eluent and further purified by gel permeation chromatography (GPC) using chloroform as eluent, to afford 245 mg (0.7 mmol) of pure L^A with an 82.5 % yield as orange powder.

ESI-MS: Found MH^+ 335.1157; Calc. 335.1179

^{1}H-NMR (500 MHz, $CDCl_3$): δ 8.91 (s, 2H, H_{IV}), 8.65 (d, J = 4.0 Hz, 2H, H_I), 7.97 (dt, J^3 = 8.0 Hz, J^4 = 2.0 Hz 2H, H_{II}), 7.94 (d, J = 1.5 Hz, 2H, H_V), 7.77 (dd, J^3 = 7.8 Hz, J^4 = 1.5 Hz, 2H, H_{VI}), 7.70 (d, J = 7.8 Hz, 2H, H_{VII}), 7.45 (dd, J^3 = 8.0 Hz, J^4 = 4.8 Hz 2H, H_{III}).

^{13}C-NMR (150 MHz, $CDCl_3$): δ 192.62 (s, 1C), 148.69 (s, 2C), 147.48 (s, 2C), 143.20 (s, 2C), 138.75 (s, 2C), 134.97 (s, 2C), 133.80 (s, 2C), 133,10 (s, 2C), 127.41 (s, 2C), 123.38 (s, 2C), 122.77 (s, 2C), 120.91 (s, 2C).

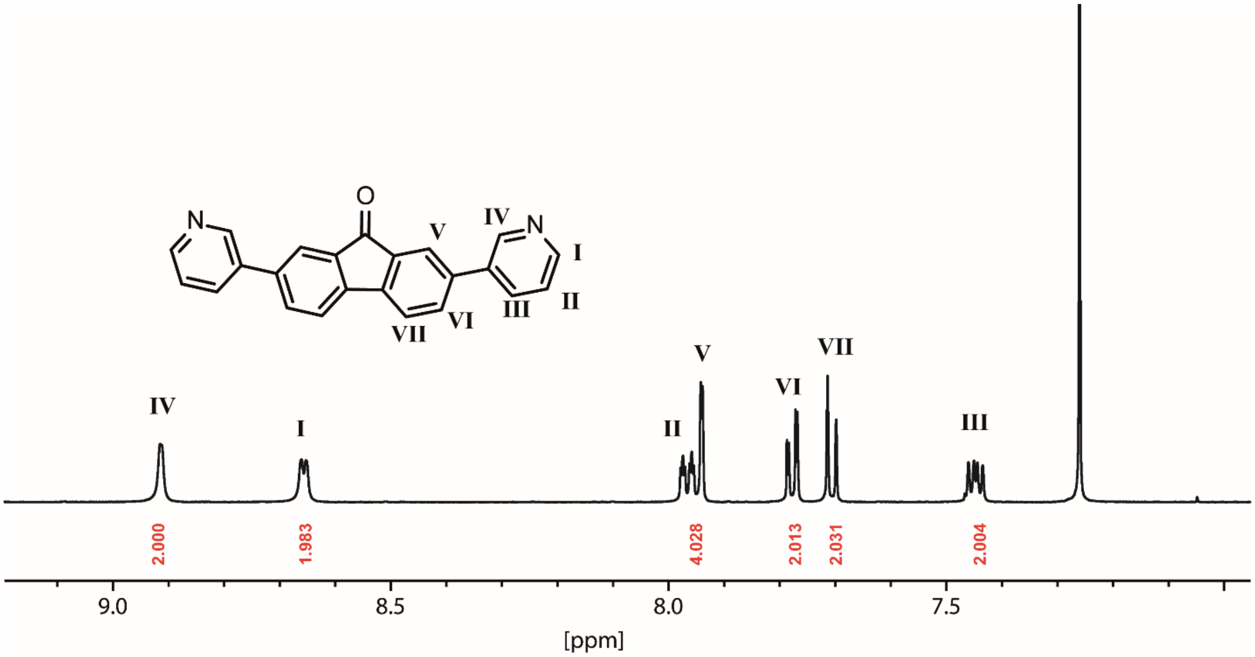

Figure S1: ^{1}H NMR spectrum (500 MHz, 298K, $CDCl_3$) of ligand L^A.

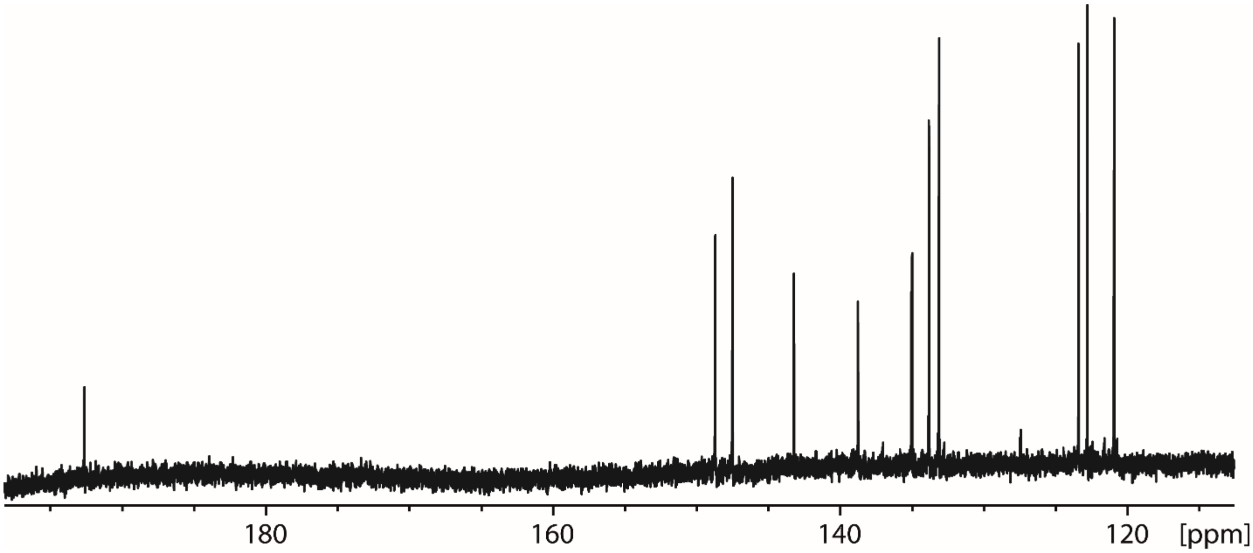

Figure S2: ^{13}C NMR spectrum (150 MHz, 298K, $CDCl_3$) of ligand L^A.

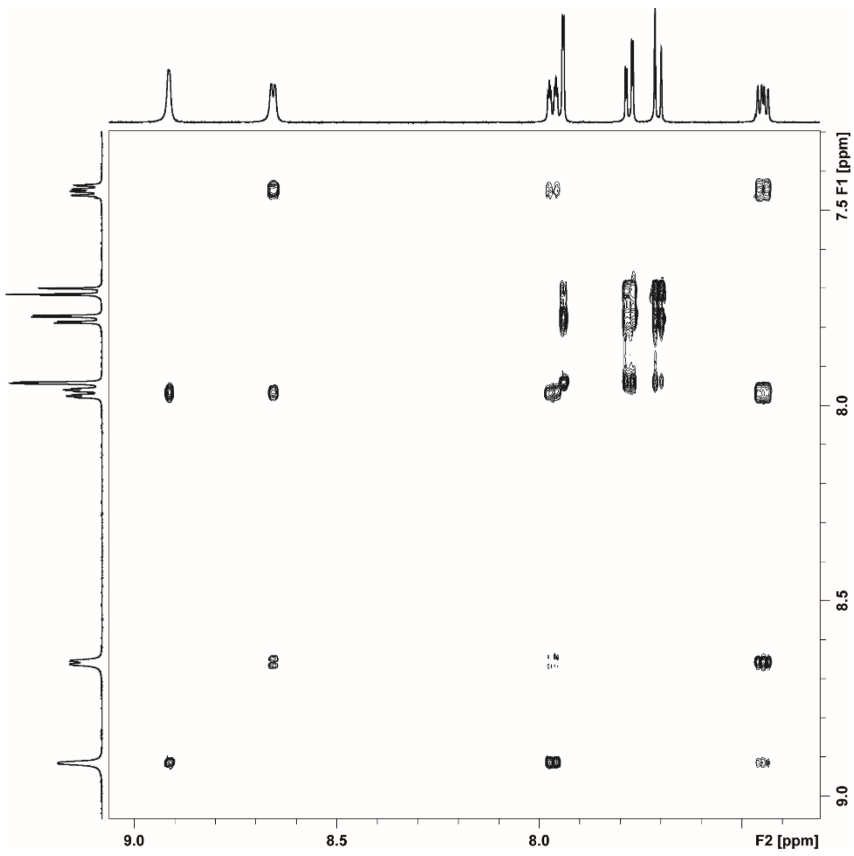

Figure S3: COSY NMR spectrum (500 MHz, 298K, $CDCl_3$) of ligand L^A.

2.1.2 Synthesis of L^B.

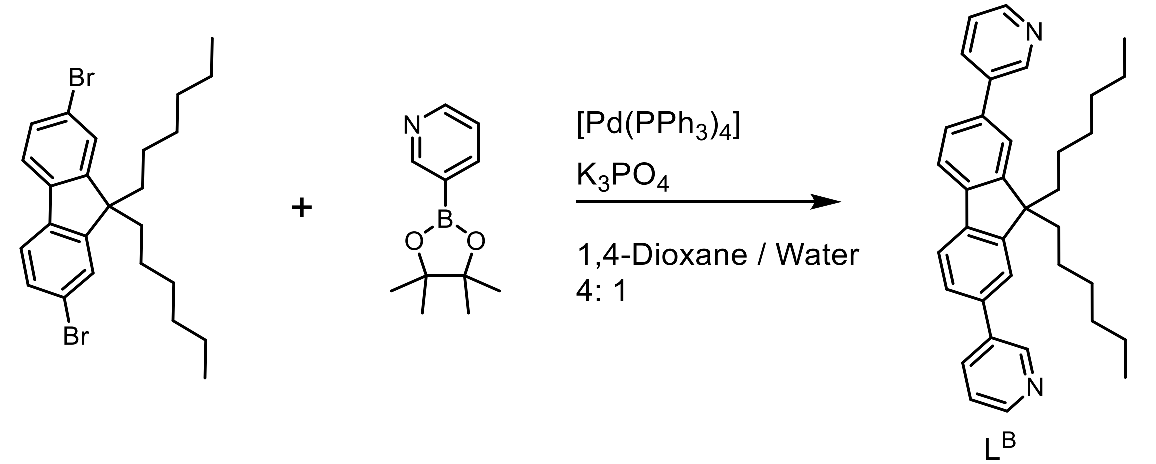

Scheme S2. Synthesis of L^B

A 3 mL aqueous solution of K_3PO_4 (180 mg, 0.85 mmol) was added to a 1,4-dioxane (12 mL) solution of 2,7-dibromo-9,9-dihexyl fluorene (200 mg, 0.4 mmol) and pyridine-3-boronic acid pinacol ester (210 mg, 1.0 mmol). The resulting solution was thoroughly degassed and $[Pd(PPh_3)_4]$ (34 mg, 0.03 mmol) were

added under nitrogen atmosphere. After stirring at 90 °C overnight, the mixture was allowed to cool down to room temperature and 20 mL of dichloromethane were added. The solution was washed with brine (3 x 15 mL) and dried over $MgSO_4$. The solvent was removed under vacuum affording a yellowish solid. The crude product was purified through column chromatography (silica) using 25 % ethyl acetate in pentane as eluent and further purified by gel permeation chromatography (GPC) using chloroform as eluent, to afford 180 mg (0.36 mmol) of pure L^B with an 91 % yield as white powder.

ESI-MS: Found MH^+ 489.3261; Calc. 489.3264

^{1}H-NMR (500 MHz, CD_3CN): δ 8.95 (s, 2H, H_a), 8.58 (s, 2H, H_b), 8.06 (dt, J^3 = 8.0 Hz, J^4 = 1.9 Hz 2H, H_d), 7.91 (d, J = 7.9 Hz, 2H, H_e), 7.76 (d, J = 1.3 Hz, 2H, H_g), 7.69 (dd, J^3 = 7.9 Hz, J^4 = 1.7 Hz 2H, H_f), 7.45 (dd, J^3 = 8.0 Hz, J^4 = 4.7 Hz 2H, H_c), 2.14, (m, 4H, alkyl chain, overlap with H_2O), 1.05 (m, 12H , alkyl chain), 0,72 (m, 6H, alky chain), 0.61 (m, 4H, alkyl chain).

^{13}C-NMR (150 MHz, CD_3CN): δ 153.08 (s, 2C), 149.36 (s, 2C), 149.05 (s, 2C), 141.59 (s, 2C), 137.95 (s, 2C), 137.57 (s, 1C), 135.19 (s, 2C), 127.13 (s, 2C), 124,74 (s, 2C), 122.75 (s, 2C), 121.51 (s, 2C), 56.58 (s, 2C), 40.66 (s, 2C), 32.10 (s, 2C), 30.13 (s, 2C), 24.62 (s, 2C), 23.15 (s, 2C), 14.20 (s, 2C).

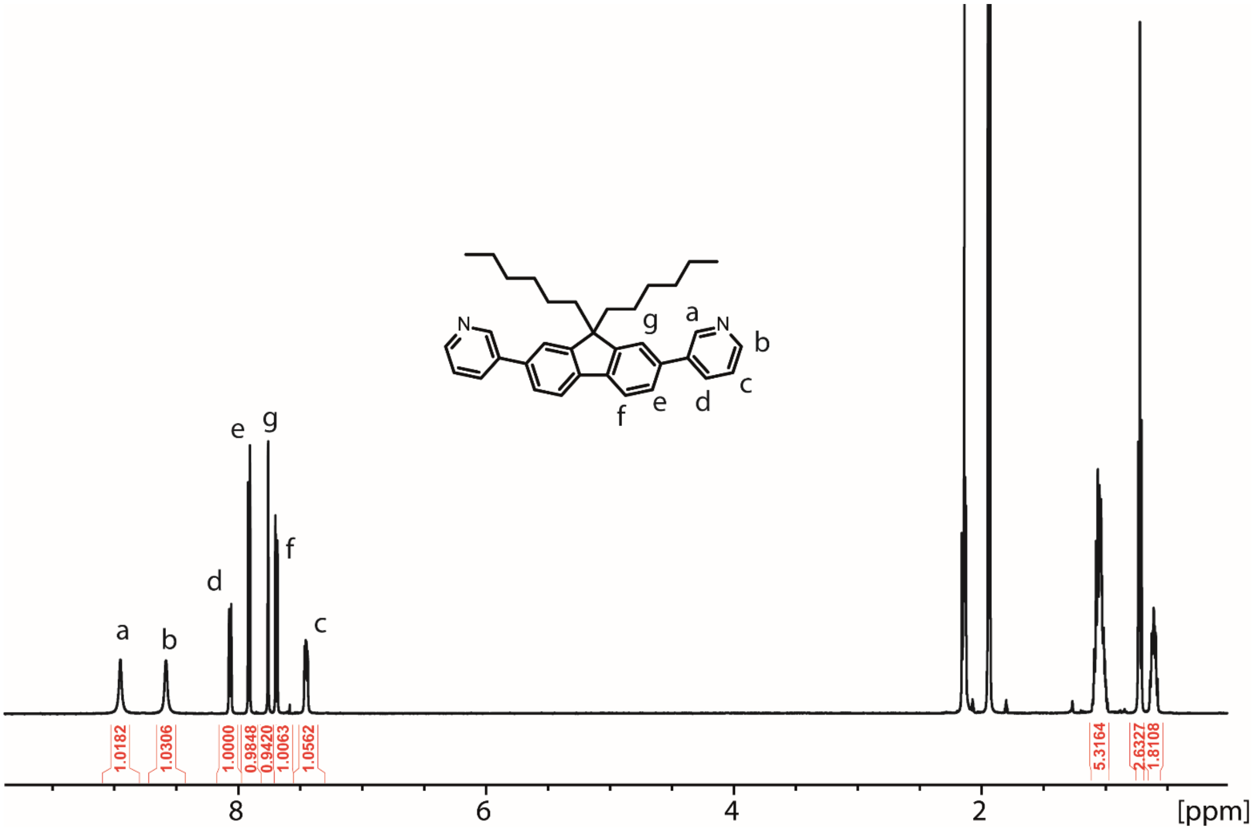

Figure S4: ^{1}H NMR spectrum (500 MHz, 298K, CD_3CN) of ligand L^B.

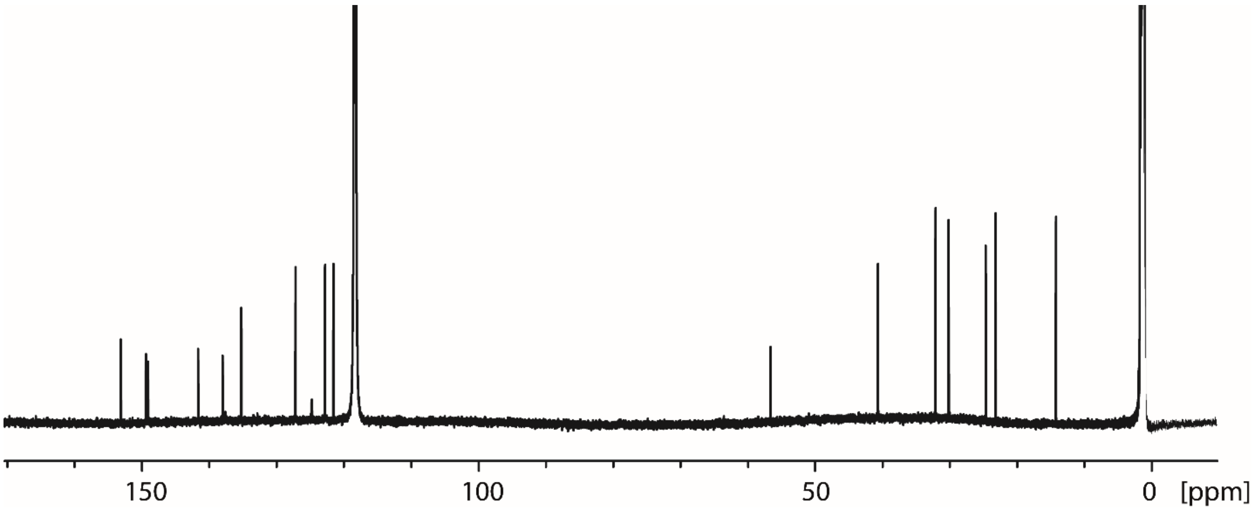

Figure S5: ^{13}C NMR spectrum (150 MHz, 298K, CD_3CN) of ligand L^B.

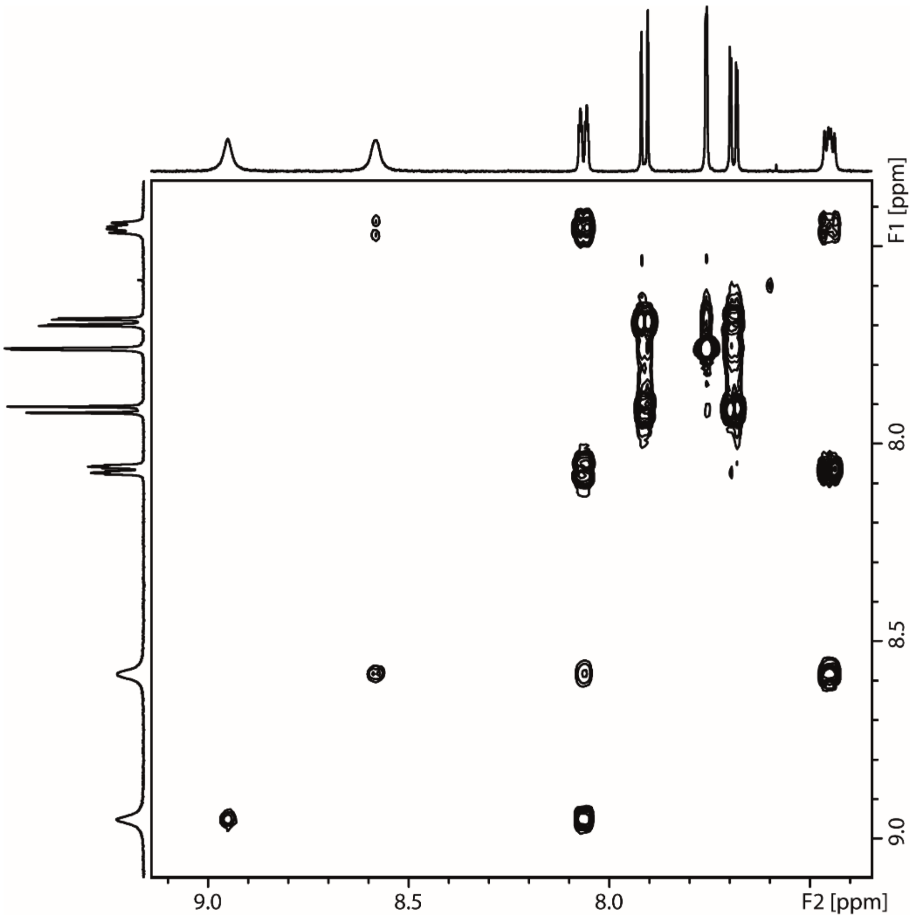

Figure S6: COSY NMR spectrum (500 MHz, 298K, CD_3CN) of ligand L^B.

2.2 Cage synthesis

2.2.1 Synthesis of $[Pd_nL^A_{2n}]$ in CD_3CN.

A solution of $[Pd(CH_3CN)_4](BF_4)_2$ (33.6 µL from a 25 mM stock solution in CD_3CN, 0.84 mmol, 1.0 eq.) was added to a solution of ligand L^A (0.57 mg, 1.68 mmol, 2.0 eq.) to afford 600 µL of a 2.8mM (ligand concentration) CD_3CN solution. After mixing the sample at RT for 1 hour and 30 minutes, or after heating to 70 °C for 30 minutes, formation of a $[Pd_nL^A_{2n}]$ (n=3,4,6) product mixture was confirmed by NMR and ESI-MS techniques.

^{1}H-NMR (500 MHz, CD_3CN): δ 9.63 (d, J = 1.8 Hz, 1H, $H_{IV''}$), 9.56 (d, J = 1.8 Hz, 1H, $H_{IV'}$), 9.48 (d, J = 2.0 Hz, 2H, H_{IV}), 9.23 (m, 3H, overlap of $H_I + H_{I'}$), 9.11 (dd, J^3 = 5.8 Hz, J^4 = 1.0 Hz, 1H, $H_{I''}$), 8.32 (dt, J^3 = 8.4 Hz, J^4 = 1.6 Hz, 1H, $H_{III''}$), 8.21 to 8.13 (m, 5H, overlap of $H_{III} + H_{III'} + H_{VI'} + H_{VII'}$), 7.97 to 7.92 (m, 4H, overlap of $H_{VI} + H_{V'} + H_{V''}$), 7.84 (dd, J^3 = 7.9 Hz, J^4 = 1.6 Hz, 2H, H_{VII}), 7.79 (d, J = 7.8 Hz, 1H, $H_{VI'}$), 7.78 to 7.60 (m, 4H, overlap of $H_{II} + H_{II''} + H_{VII'}$), 7.67 to 7.63 (m, 3H, overlap of $H_V + H_{II}$).

Signals assigned to $[Pd_3L^A_6]$ = I, II, III, IV, V, VI, VII; Signals assigned to $[Pd_4L^A_8]$ = I', I'', II', II'', III', III'', IV', IV'', V', V'', VI', VI'', VII', VII''.

^{13}C-NMR (150 MHz, CD_3CN): δ 192.88 (s, 1C), 172.99 (s, 2C), 150.88 (s, 2C), 150.63 (s, 2C), 150.40 (s, 2C), 149.69 (s, 2C), 149.42 (s, 2C), 145.53 (s, 2C), 145.31 (s, 2C), 140.83 (s, 2C), 140.56 (s, 2C), 140.22 (s, 2C), 139.83 (s, 2C), 139.63 (s, 2C), 137.76 (s, 2C), 137.44 (s, 2C), 137.06 (s, 2C), 136.95 (s, 2C), 136.63 (s, 2C), 136.42 (s, 2C), 135.63 (s, 2C), 135.42 (s, 2C), 135.17 (s, 2C), 128.67 (s, 2C), 128.54 (s, 2C), 128.29 (s, 2C), 123.53 (s, 2C), 123.45 (s, 2C), 123.39 (s, 2C), 123.32 (m, 4C), 122.93 (s, 2C). Low solubility of the components of the compound mixture results in weak signals, hiding 4 signals of $[Pd_4L^A_8]$.

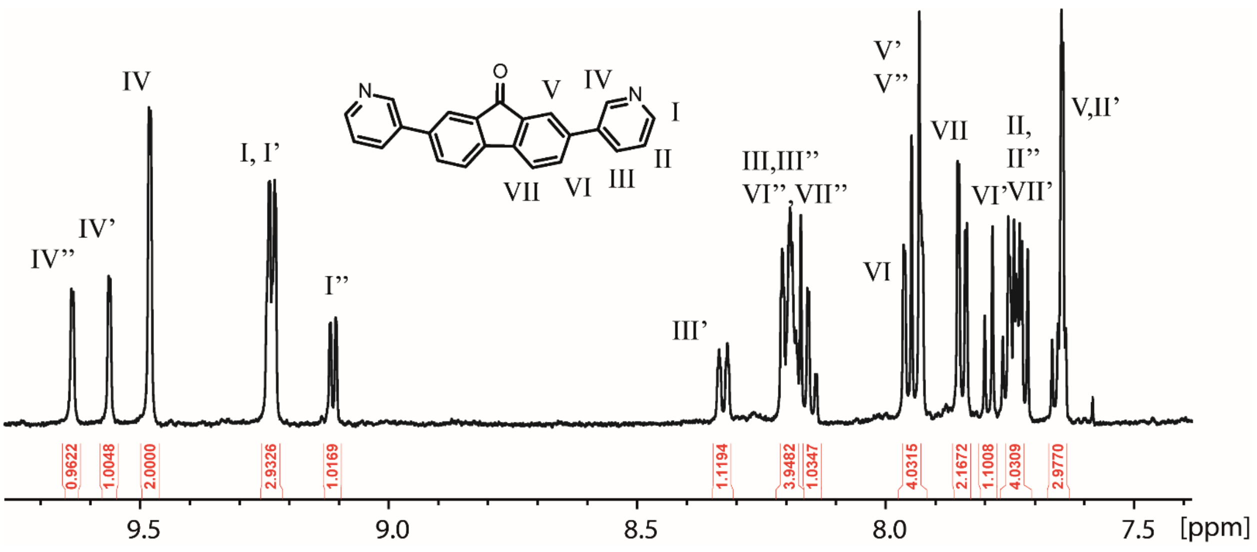

Figure S7: ^{1}H NMR spectrum (500 MHz, 298K, CD_3CN) of [$Pd_nL^{A}_{2n}$].

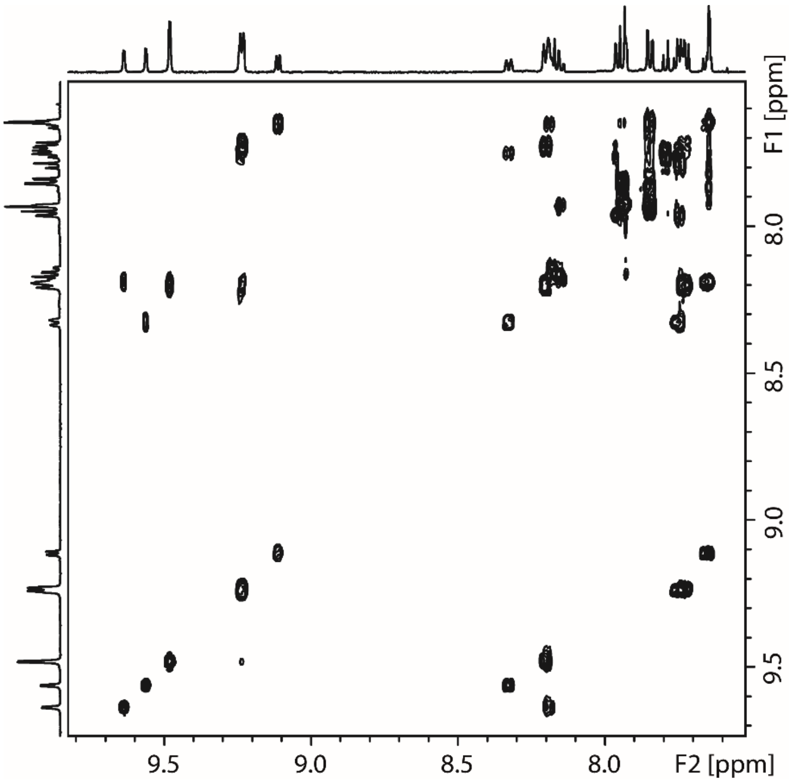

Figure S8: COSY NMR spectrum (500 MHz, 298K, CD_3CN) of [$Pd_nL^{A}_{2n}$].

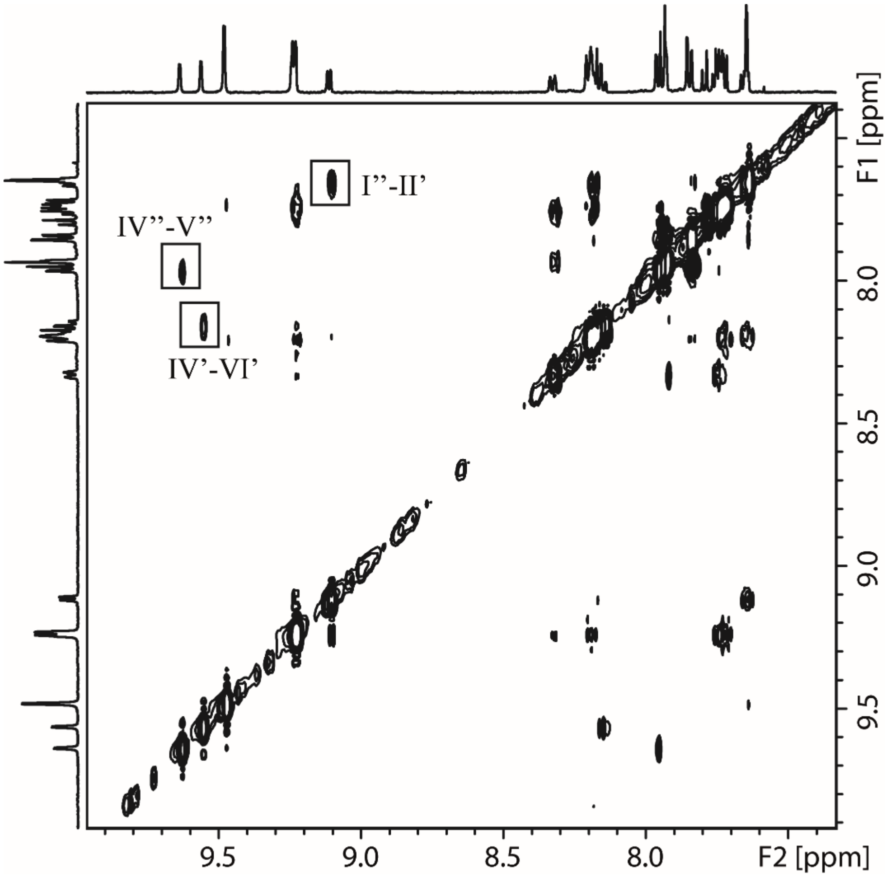

Figure S9: NOESY NMR spectrum (500 MHz, 298K, CD_3CN) of $[Pd_nL^A_{2n}]$.

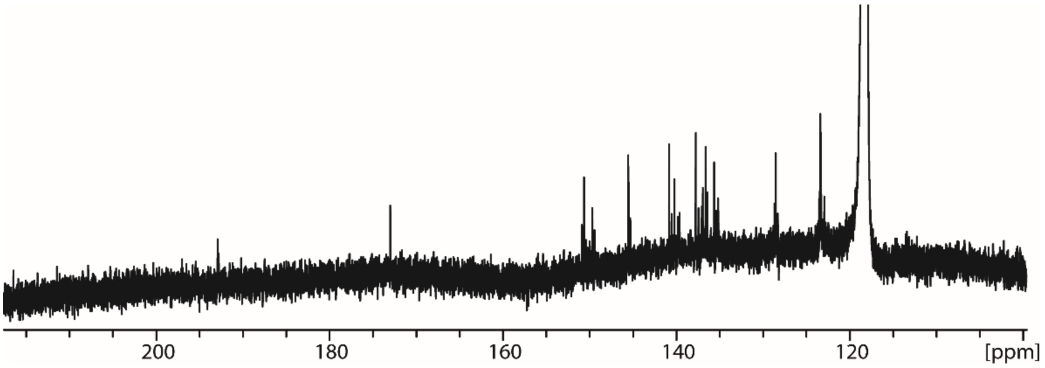

Figure S10: ^{13}C NMR spectrum (150 MHz, 298K, CD_3CN) of $[Pd_nL^A_{2n}]$, low signal intensity and missing signals are due to low solubility of the components of the compound mixture.

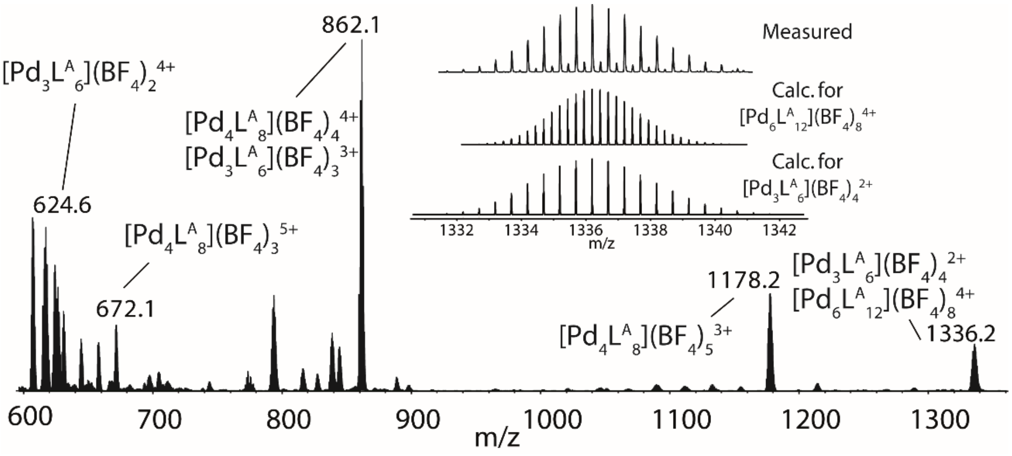

Figure S11: ESI-MS spectrum of $[Pd_nL^A_{2n}]$ in CH_3CN, with isotopic pattern for the signals of $[Pd_3L^A_6(BF_4)_4]^{2+}$ and $[Pd_6L^A_{12}(BF_4)_8]^{4+}$ shown in the inset.

2.2.2 Synthesis of $[Pd_3L^A_6]$ in DMSO-d_6.

A solution of $[Pd(CH_3CN)_4](BF_4)_2$ (33.6 µL from a 25 mM stock solution in DMSO-d_6, 0.84 mmol, 1.0 eq.) was added to a solution of ligand L^A (0.57 mg, 1.68 mmol, 2.0 eq.) to afford 600 µL of a 2.8mM (ligand concentration) DMSO-d_6 solution. After mixing the sample at RT for 1 hour and 30 minutes, or after heating to 70 °C for 30 minutes, formation of $[Pd_3L^A_6]$ was confirmed by NMR and ESI-MS techniques.

^{1}H-NMR (500 MHz, DMSO-d_6): δ 9.67 (s, 2H, H_{IV}), 9.49 (d, J = 5.7 Hz, 2H, H_I), 8.41 (d, J = 8.2 Hz, 2H, H_{III}), 8.12 (d, J = 7.7 Hz, 2H, H_{VI}), 8.07 (d, J = 7.7 Hz, 2H, H_{VII}), 7.91 (dd, J = 8.2 Hz, J = 5.7 Hz, 2H, H_{II}), 7,56 (s, 2H, H_V). * = signals assigned to $[Pd_4L^A_8]$.

^{13}C-NMR (150 MHz, DMSO-d_6): δ 191.34 (s, 1C), 171.41 (s, 2C), 150.03 (s, 2C), 148.48 (s, 2C), 144.13 (s, 2C), 139.24 (s, 2C), 138.47 (s, 2C), 136.45 (s, 2C), 135.22 (s, 2C), 134.65 (s, 2C), 127.47 (s, 2C), 122.84 (s, 2C), 121.82 (s, 2C).

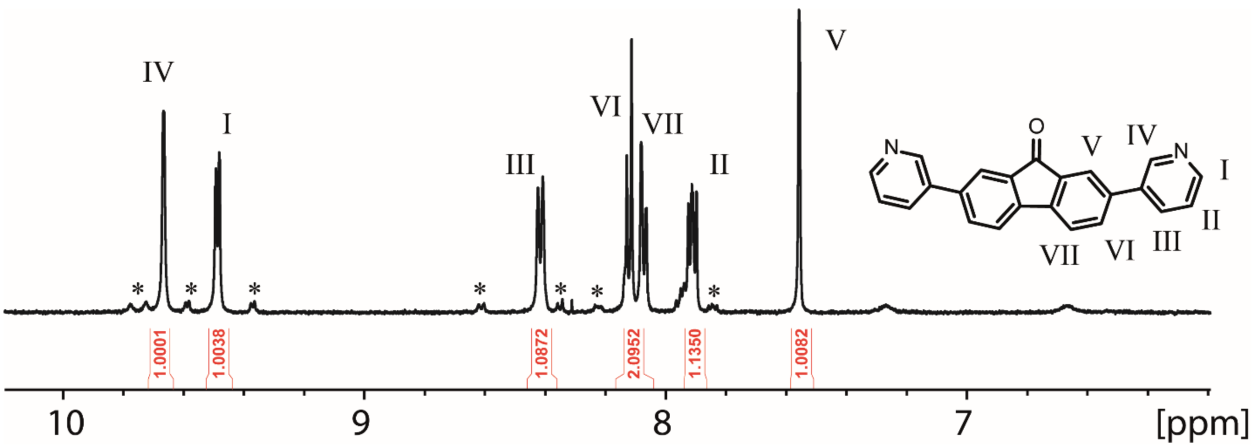

Figure S12: ^{1}H NMR spectrum (500 MHz, 298K, DMSO-d_6) of [$Pd_3L^A{}_6$], * = signals of [$Pd_4L^A{}_8$].

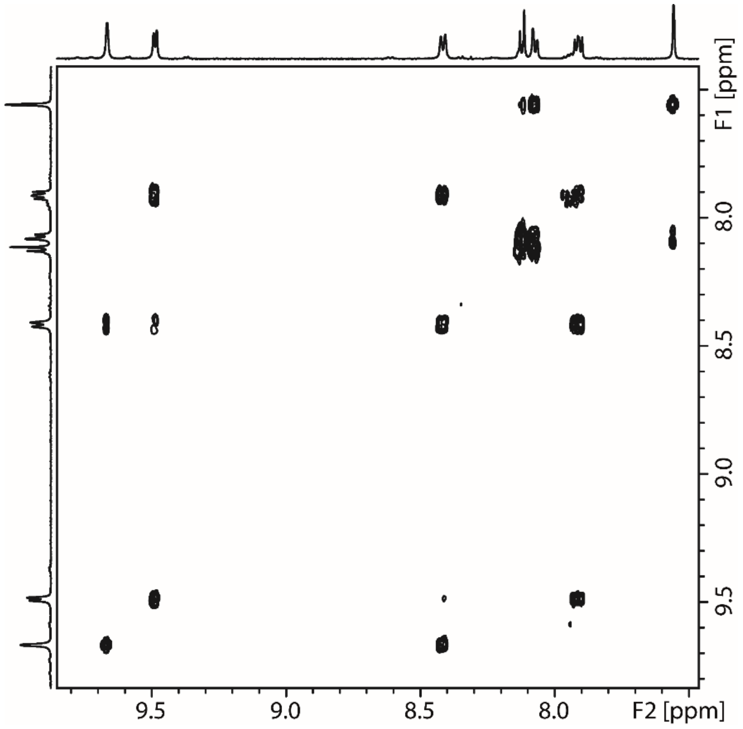

Figure S13: COSY NMR spectrum (500 MHz, 298K, DMSO-d_6) of [$Pd_3L^A{}_6$].

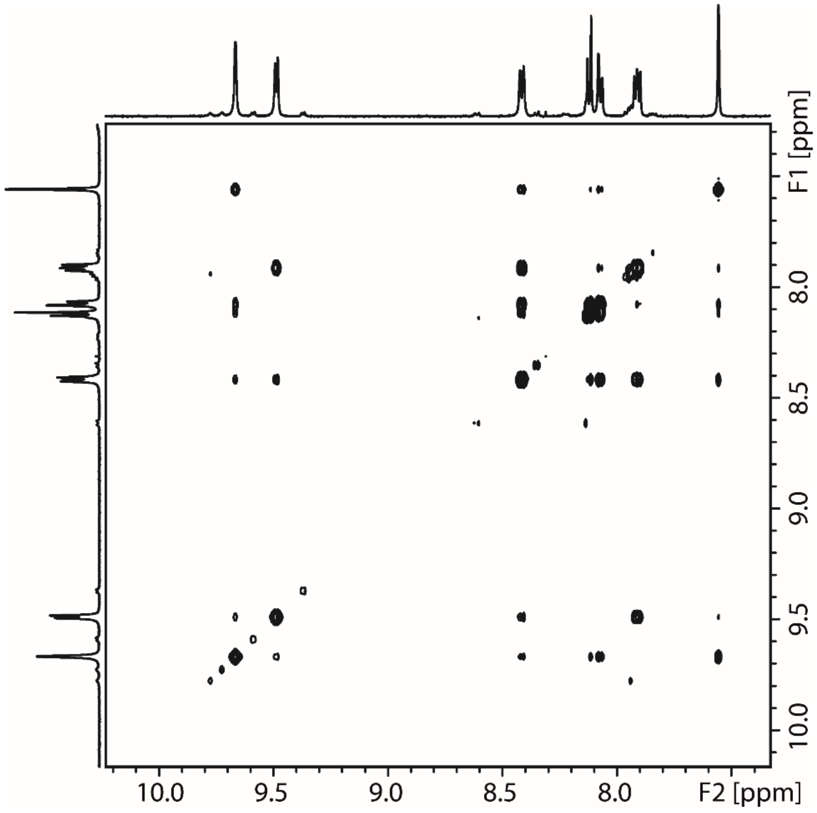

Figure S14: NOESY NMR spectrum (500 MHz, 298K, DMSO-d_6) of [$Pd_3L^A{}_6$].

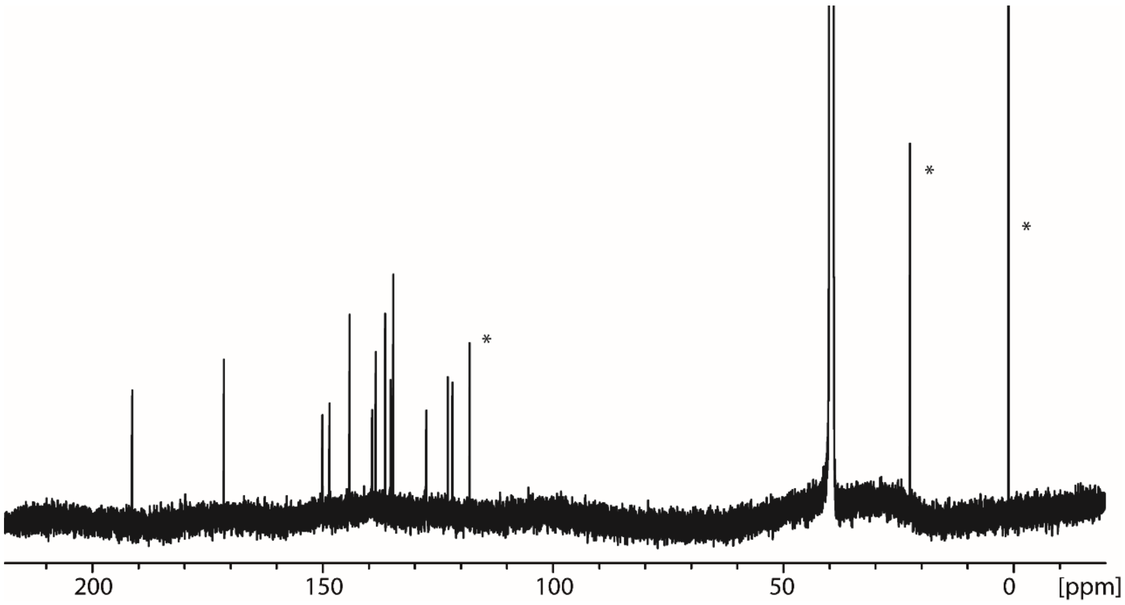

Figure S15: ^{13}C NMR spectrum (150 MHz, 298K, DMSO-d_6) of [$Pd_3L^A{}_6$]. *CH_3CN and residual solvent.

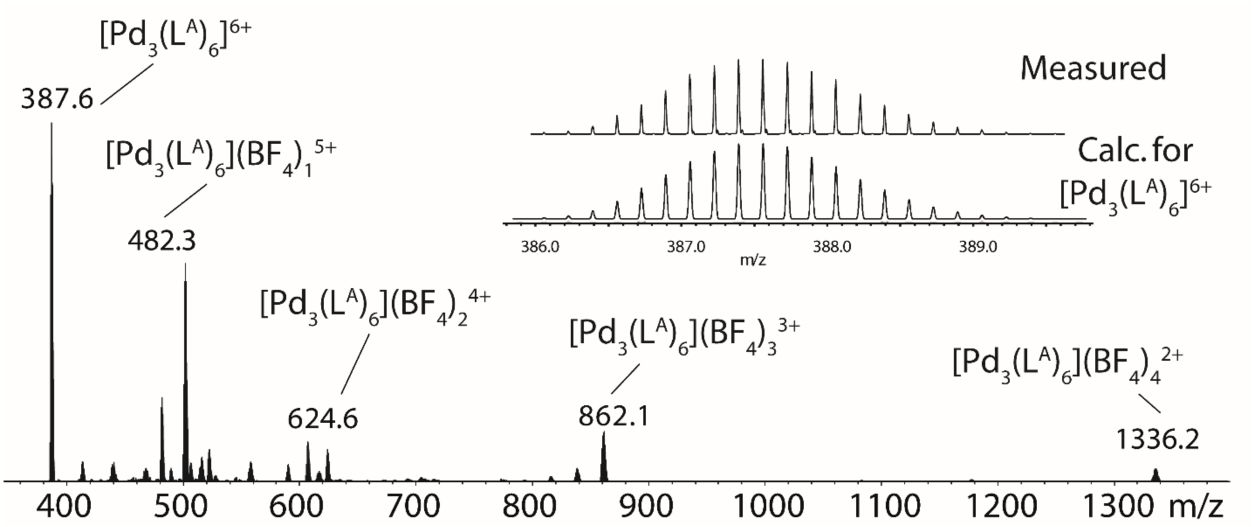

Figure S16: ESI-MS spectrum of $[Pd_3L^A_6]$ in DMSO, with isotopic pattern for the signal of $[Pd_3L^A_6]^{6+}$ shown in the inset.

2.2.3 Synthesis of $[Pd_6L^B_{12}]$.

A solution of $[Pd(CH_3CN)_4](BF_4)_2$ (33.6 µL from a 25 mM stock solution in either CD_3CN or DMSO-*d₆*, 0.84 mmol, 1.0 eq.) was added to a solution of ligand L^B (0.82 mg, 1.68 mmol, 2.0 eq.) to give 600 µL of either CD_3CN or DMSO-d_6 (2.8 mM in ligand concentration), respectively. After heating the sample to 70 °C for 1 hour, formation of $[Pd_6L^B_{12}]$ was confirmed by NMR and ESI-MS techniques.

^{1}H-NMR (500 MHz, CD_3CN δ 9.60 (d, J = 1.4 Hz, 2H, H_a), 8,92 (d, J = 5.5 Hz, 2H, H_b), 8.28 (d, J = 8.2 Hz, 2H, H_d), 8.19 (dd, J^3 = 8.1 Hz, J^4 = 1.4 Hz 2H, H_e), 8.11 (d, J = 8.1 Hz, 2H, H_f), 7.65 (s, 2H, H_g), 7.61 (dd, J = 8.2 Hz, J = 5.5 Hz, 2H, H_c), 1.98 (m, br , 4H, alkyl chain overlapped with solvent), 0.85 to 0.68 (m, br, 12H, alkyl chain), 0.44 to 0.31 (m, br, 10H, alkyl chain).

^{1}H-NMR (500 MHz, DMSO-d_6): δ 9.72 (s, br, 2H, H_a), 9.43 (dd, J = 5.1 Hz, 2H, H_b), 8.54 (d, J = 7.9 Hz, 2H, H_d), 8.19 (d, J = 8.0 Hz, 2H, H_e), 8.13 (d, br, J = 8.0 Hz, 2H, H_f), 7.94 (s, 2H, H_g), 7.86 (m, 2H, H_c), 1.98 (s, br , 4H, alkyl chain), 0.8 to 0.62 (m, br, 12H, alkyl chain), 0.37 to 0.27 (m, br, 10H, alkyl chain).

^{13}C-NMR (150 MHz, DMSO-d_6): δ 171.43 (s, 2C), 152.27 (s, 2C), 149.32 (s, 2C), 148.17 (s, 2C), 141.12 (s, 2C), 138.26 (m, 4C), 133.14 (s, 1C), 127.29 (s, 2C), 125.28 (s, 2C), 121.60 (s, 2C), 121.14 (s, 2C), 55.22 (s, 2C), 30.44 (s, 2C), 28.56 (s, 2C), 23.07 (s, 2C), 21.56 (s, 2C), 13.34 (s, 2C).

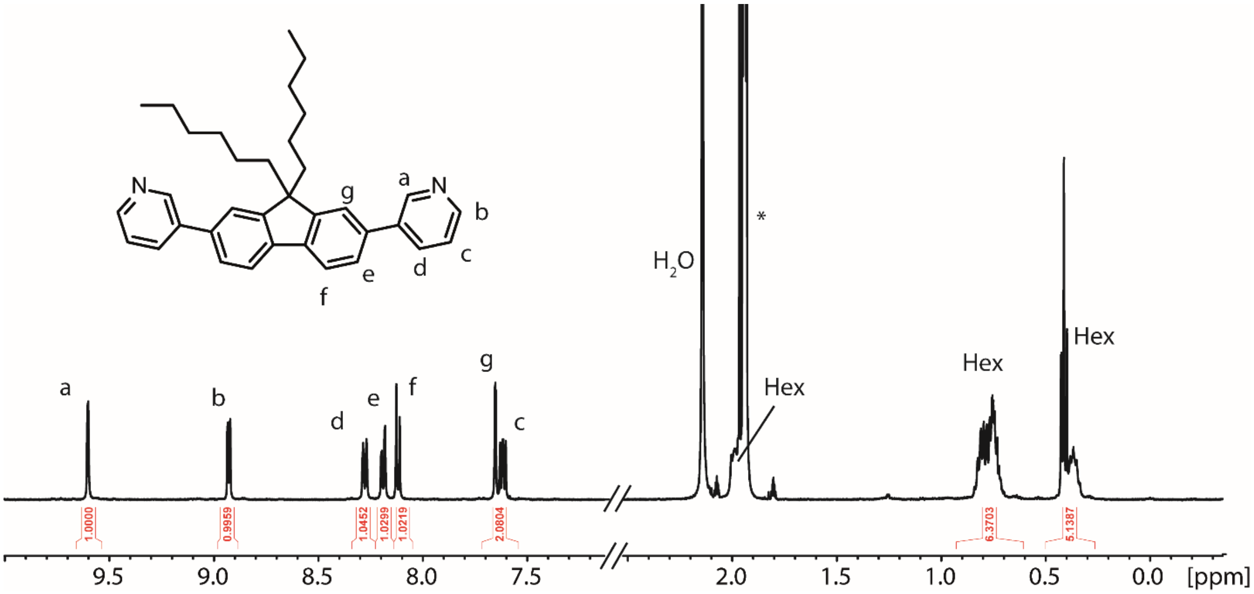

Figure S17: ^1H NMR spectrum (500 MHz, 298K, CD_3CN) of [$Pd_6L^B{}_{12}$], Hex = signals of the alkyl chains, * = signals of solvent and CH_3CN.

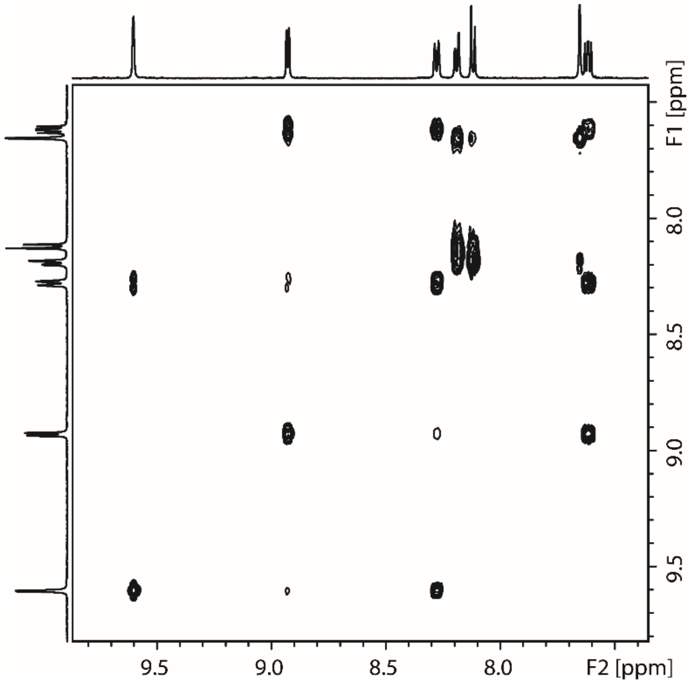

Figure S18: COSY NMR spectrum (500 MHz, 298K, CD_3CN) for the aromatic region of [$Pd_6L^B{}_{12}$].

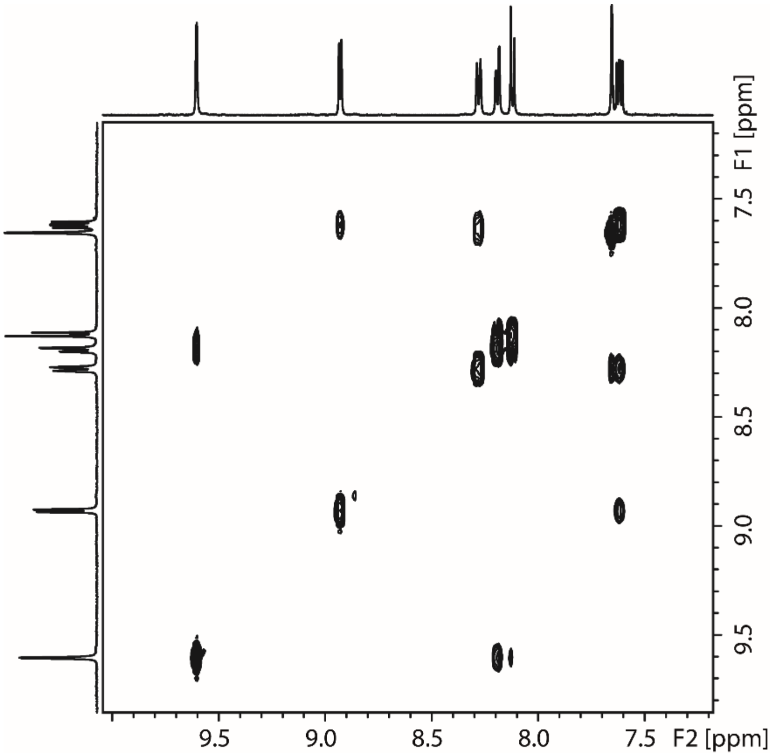

Figure S19: NOESY NMR spectrum (500 MHz, 298K, CD_3CN) for the aromatic region of [$Pd_6L^B{}_{12}$].

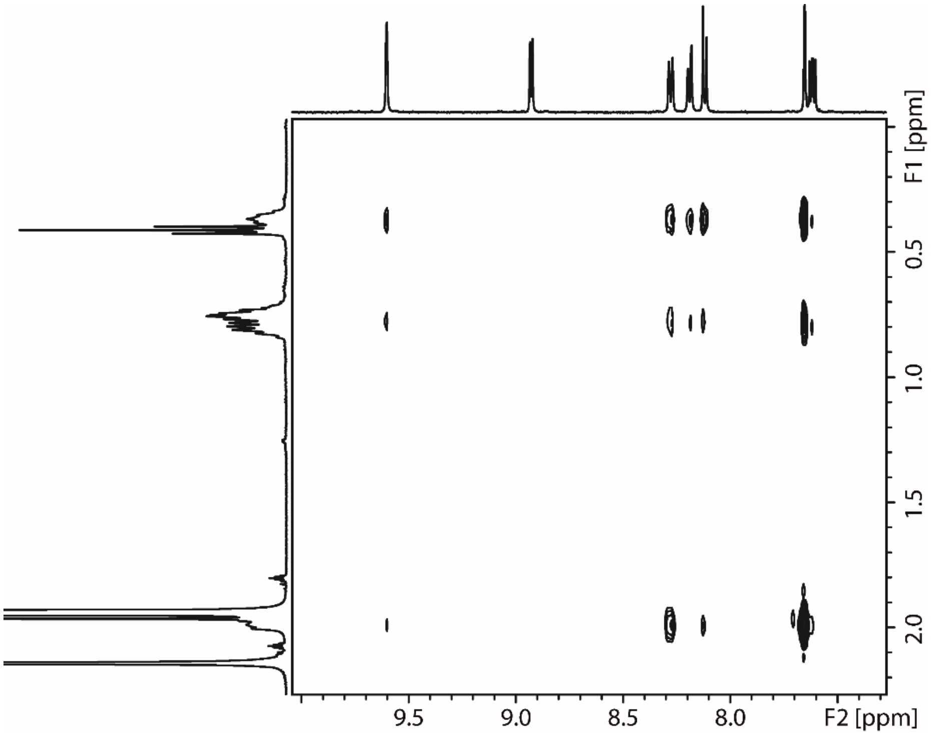

Figure S20: NOESY NMR spectrum (500 MHz, 298K, CD_3CN) for the aromatic vs aliphatic regions of [$Pd_6L^B{}_{12}$].

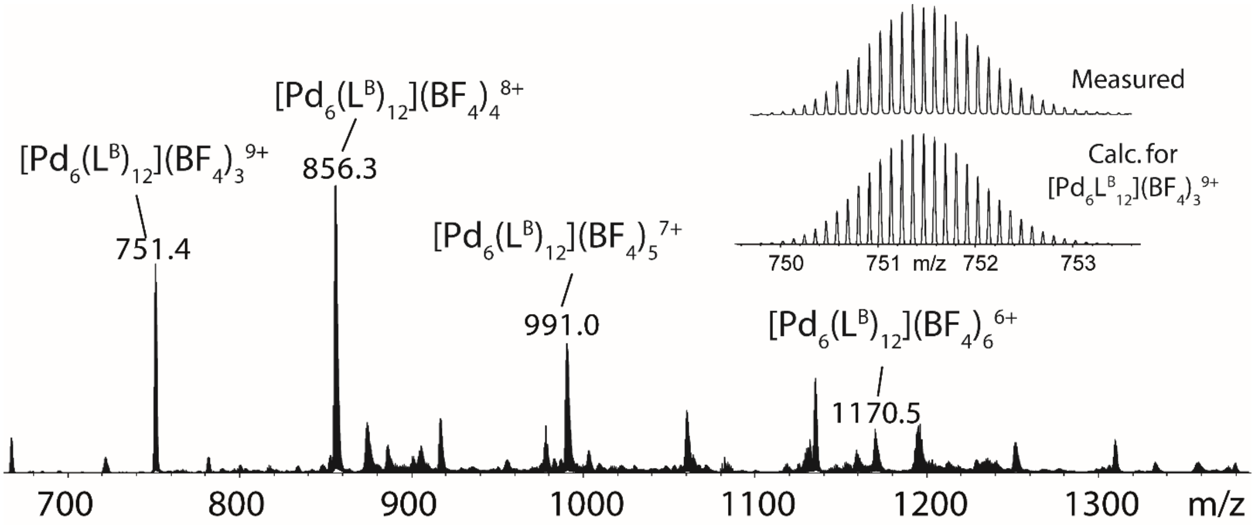

Figure S21: ESI-MS spectrum of [$Pd_6L^B{}_{12}$] assembled in CH_3CN or in DMSO afford the same identical result. Isotopic pattern for the signal of [$Pd_6L^B{}_{12}(BF_4)_3$]$^{9+}$ shown in the inset.

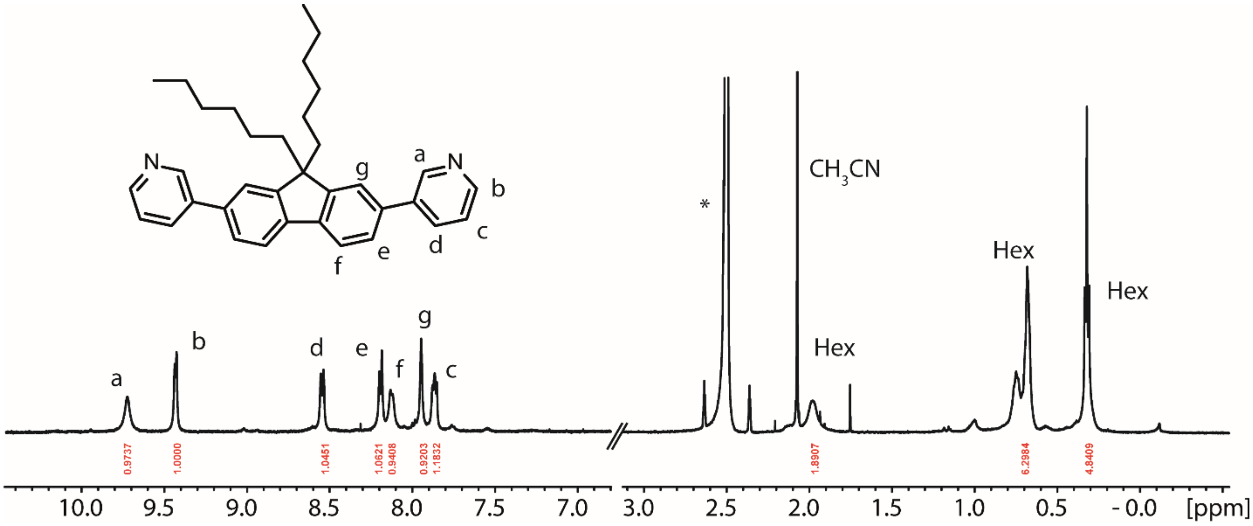

Figure S22: ^{1}H NMR spectrum (500 MHz, 298K, DMSO-d_6) of [$Pd_6L^B{}_{12}$], Hex = signals of the alkyl chains, * = solvent.

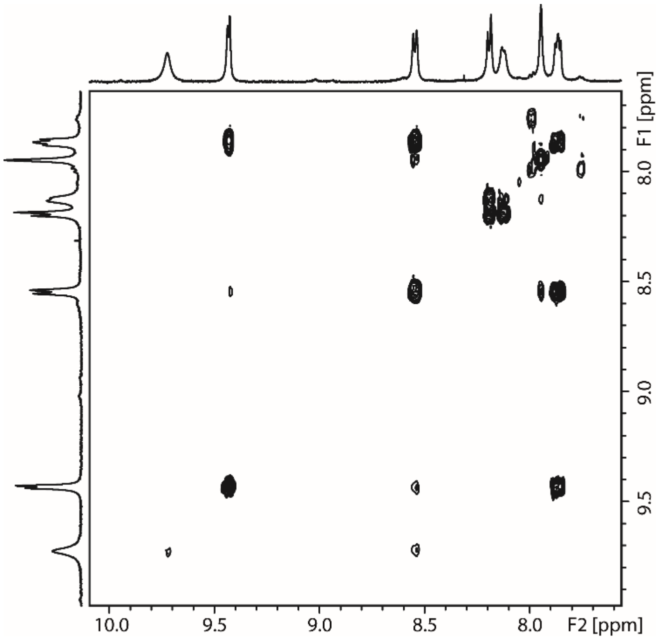

Figure S23: COSY NMR spectrum (500 MHz, 298K, DMSO-d_6) for the aromatic region of [$Pd_6L^B{}_{12}$].

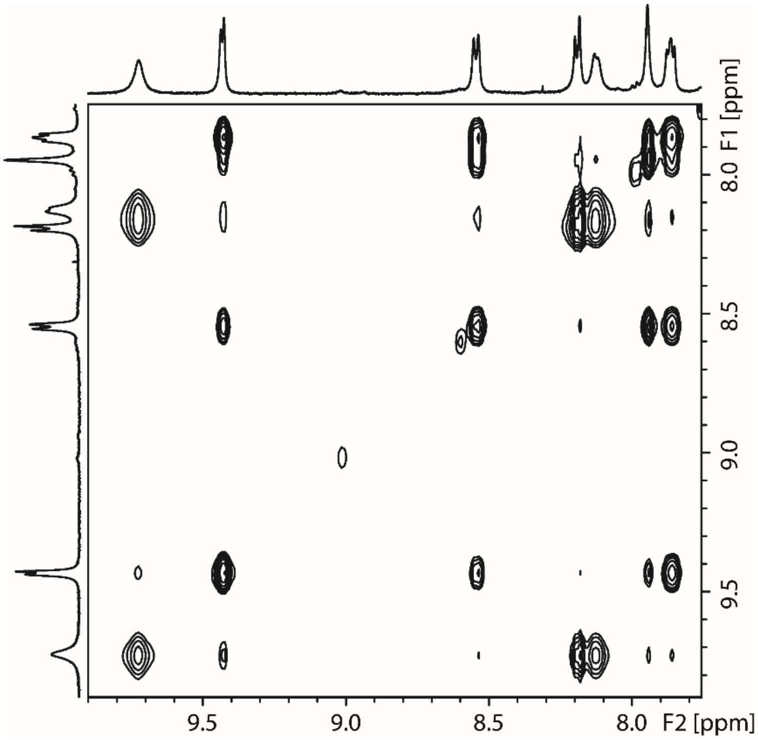

Figure S24: NOESY NMR spectrum (500 MHz, 298K, DMSO-d_6) for the aromatic region of [$Pd_6L^B{}_{12}$].

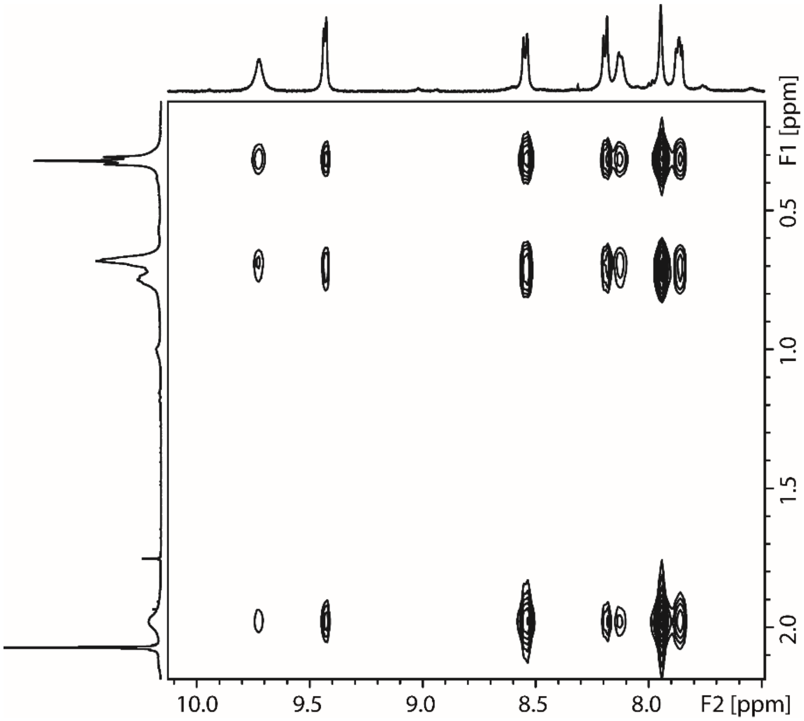

Figure S25: NOESY NMR spectrum (500 MHz, 298K, DMSO-d_6) for the aromatic vs aliphatic regions of $[Pd_6L^B{}_{12}]$.

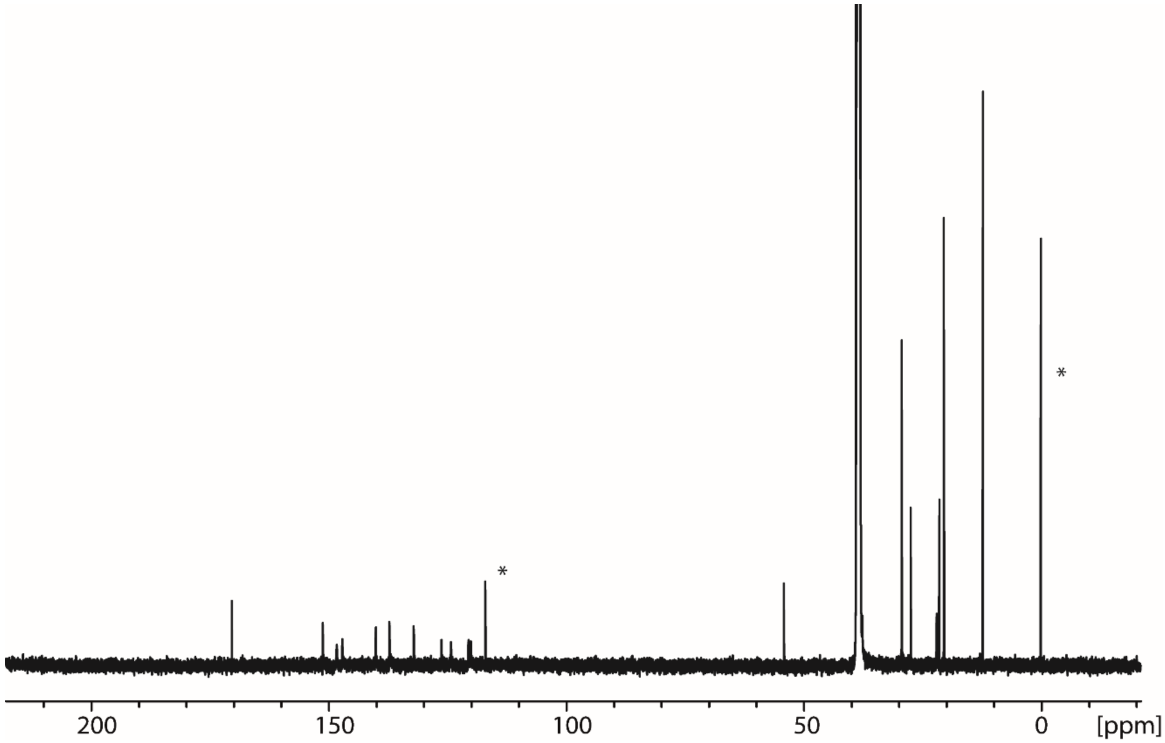

Figure S26: ^{13}C NMR spectrum (150 MHz, 298K, DMSO-d_6) of $[Pd_6L^B{}_{12}]$. *CH_3CN.

2.2.4 Synthesis of [$Pd_4L^A{}_4L^B{}_4$].

A 500 µL DMSO-d_6 solution of L^A (250 µL from a 2.8 mM solution, 1.0 eq.) and L^B (250 µL from a 2.8 mM solution, 1.0 eq.) was mixed with [$Pd(CH_3CN)_4$](BF_4)$_2$ (28.0 µL from a 25 mM stock solution in DMSO-d_6, 1.0 eq.). Heating the sample at 70 °C for 1 hour 30 minutes afforded a solution of [$Pd_4L^A{}_4L^B{}_4$], as confirmed by NMR and ESI-MS analysis.

^{1}H-NMR (500 MHz, DMSO-d_6): δ 9.85 (s, br, 2H, H_a), 9,82 (s, br, 2H, H_{IV}), 9.58 (d, J = 5.1, 2H, H_b), 9.39 (d, J = 5.4 Hz, 2H, H_I), 8.60 (d, J = 7.7 Hz, 2H, H_d), 8.42 (d, J = 8.1 Hz, 2H, H_{III}), 8.34 (d, J = 7.8 Hz, 2H, H_e), 8.05 (m, br, 4H, H_f + H_g), 7.99 to 7.89 (m, br, 8H, H_V + H_c + H_{VI} +H_{VII}), 7.86 (dd, J = 8.1 Hz, J = 5.4 Hz, 2H, H_{II}), 2.08 (m, br , 4H, alkyl chain, overlap with CH_3CN), 1.1 to 0.27 (series of m, br, 22H, alkyl chain).

^{13}C-NMR (150 MHz, DMSO-d_6): δ 191.75 (s, 1C), 171.42 (s, 4C), 152.45 (s, 2C), 150.05 (s, 2C), 149.24 (s, 2C), 148.40 (m, 4C), 143.77 (s, 2C), 141.30 (s, 2C), 138.59 (s, 2C), 138.08 (s, 2C), 137.81(s, 2C), 135.99 (s, 2C), 134.94 (m, 4C), 133.23 (s, 1C), 127.56 (s, 2C), 127.22 (s, 2C), 125.23 (s, 2C), 122.87 (s, 2C), 121.68 (s, 2C), 121.22 (m, 4C), 55.32 (s, 2C), 30.59 (s, 2C), 28.68 (s, 2C), 23.15 (s, 2C), 21.73 (s, 2C), 13.53 (s, 2C).

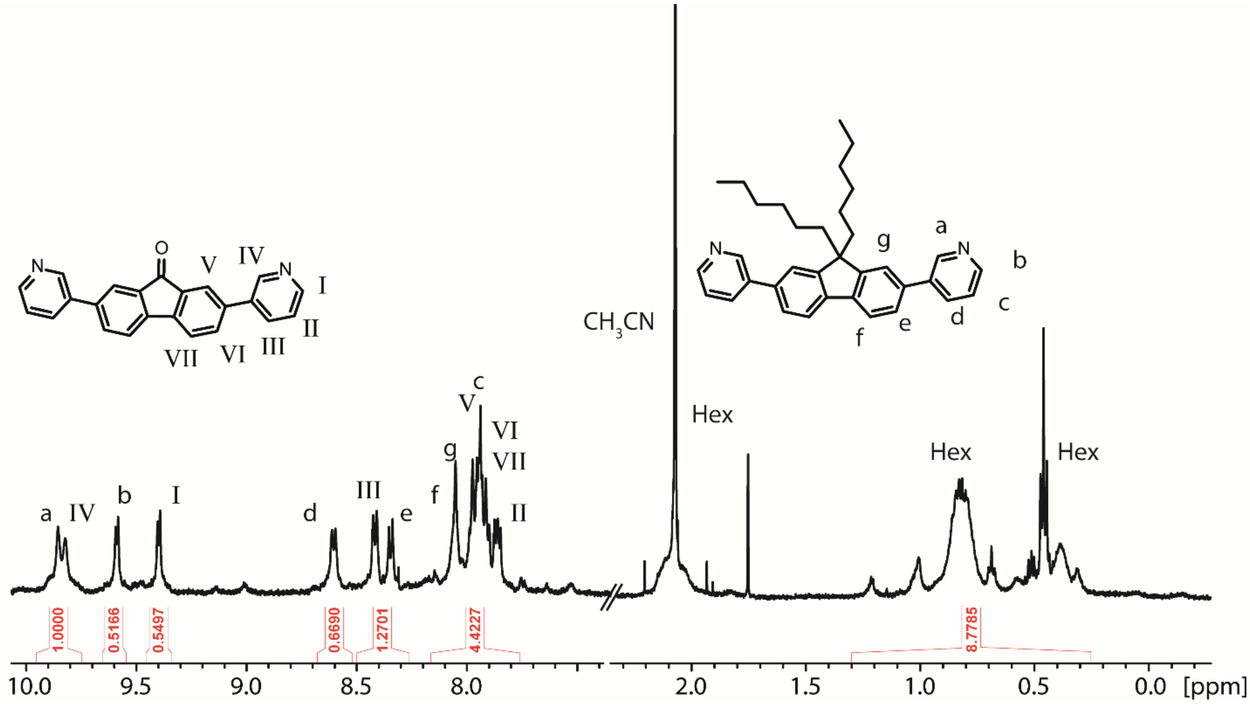

Figure S27: ^{1}H NMR spectrum (500 MHz, 298K, DMSO-d_6) of [$Pd_4L^A{}_4L^B{}_4$], Hex = signals of the alkyl chains.

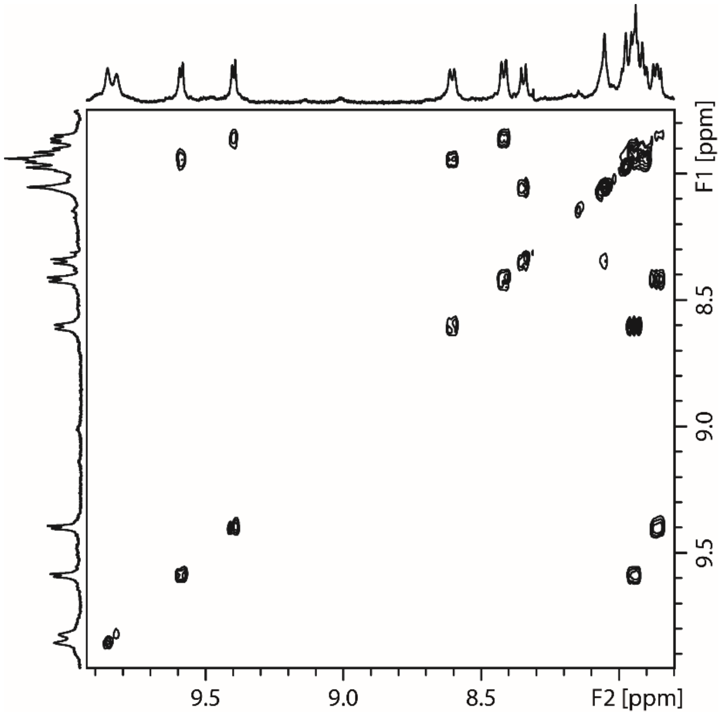

Figure S28: COSY NMR spectrum (500 MHz, 298K, DMSO-d_6) for the aromatic region of $[Pd_4L^A{}_4L^B{}_4]$.

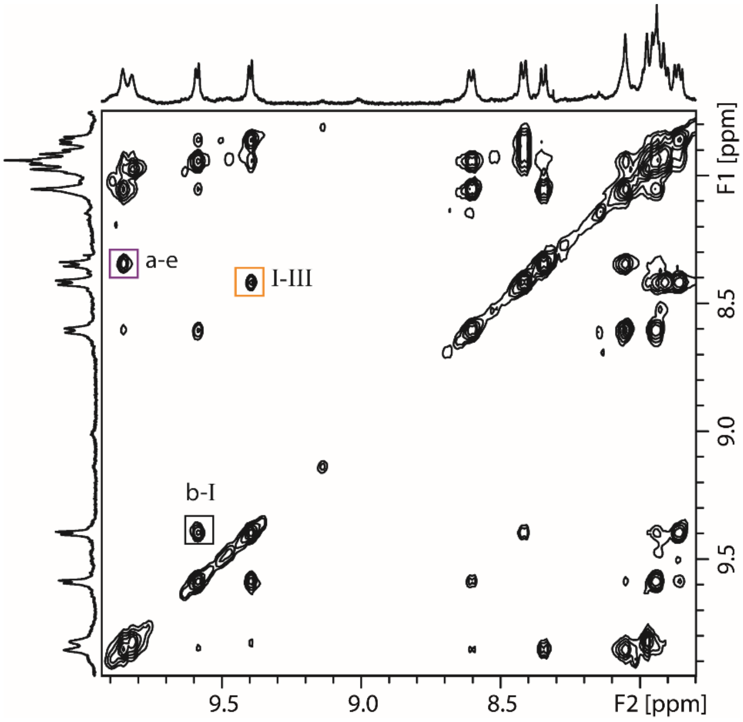

Figure S29: NOESY NMR spectrum (500 MHz, 298K, DMSO-d_6) for the aromatic region of $[Pd_4L^A{}_4L^B{}_4]$.

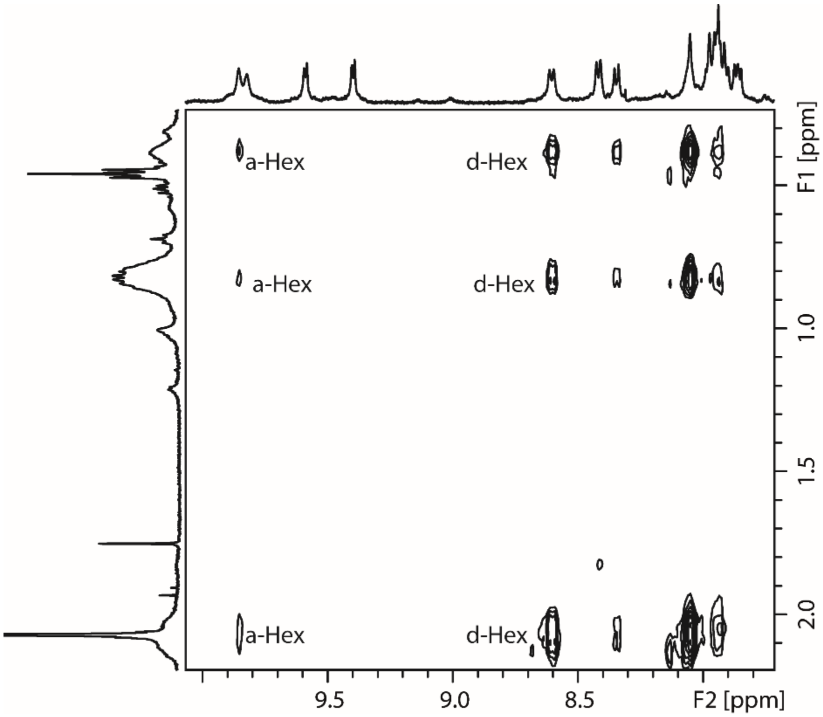

Figure S30: NOESY NMR spectrum (500 MHz, 298K, DMSO-d_6) for the aromatic vs aliphatic regions of [$Pd_4L^A{}_4L^B{}_4$].

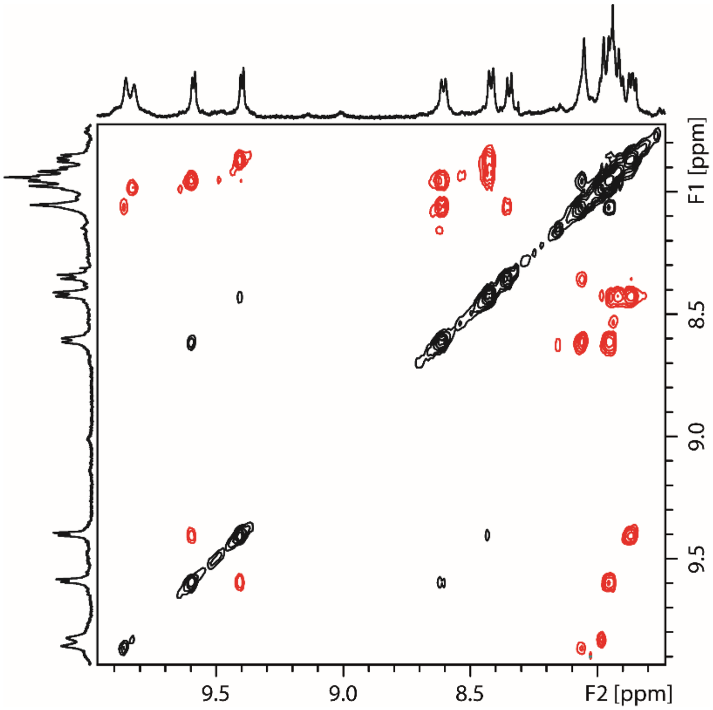

Figure S31: ROESY NMR spectrum (500 MHz, 298K, DMSO-d_6) for the aromatic region of [$Pd_4L^A{}_4L^B{}_4$].

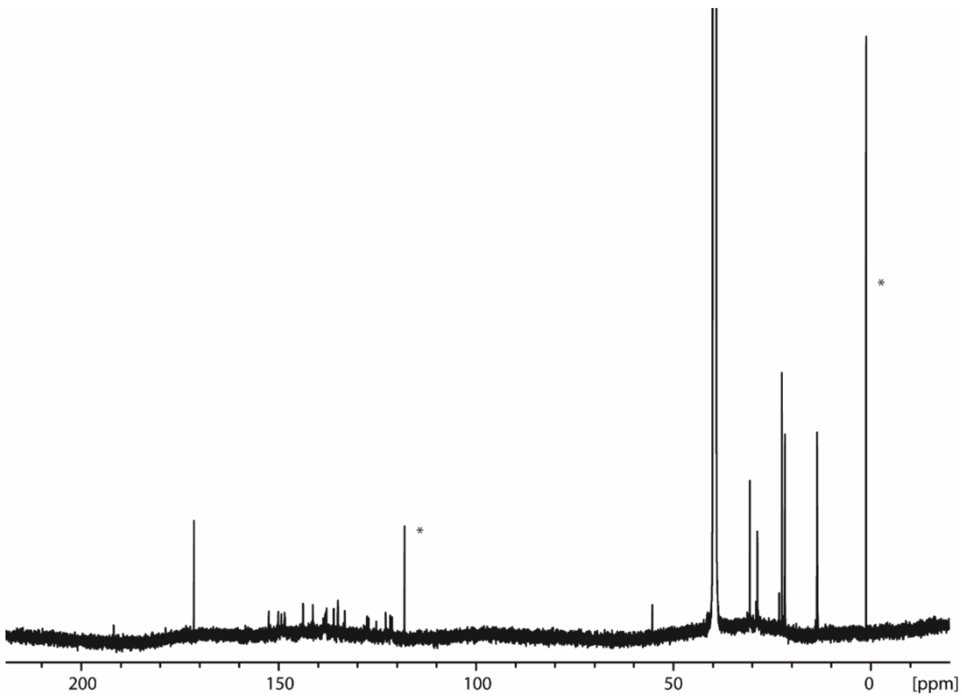

Figure S32: ^{13}C NMR spectrum (150 MHz, 298K, DMSO-d_6) of $[Pd_4L^A{}_4L^B{}_4]$. *CH_3CN; low signal intensity due to low solubility of the compound.

2.2.5 Cage to Cage transformation.

250 µL of a $[Pd_3L^A{}_6]$ 0.46 mM (2.8 mM referring to ligand concentration) DMSO-d_6 solution, was mixed with 250 µL of a $[Pd_6L^B{}_{12}]$ 0.23 mM (2.8 mM referring to ligand concentration) DMSO-d_6 solution. Heating of the sample at 70 °C overnight afforded a solution of $[Pd_4L^A{}_4L^B{}_4]$, as confirmed by NMR analysis.

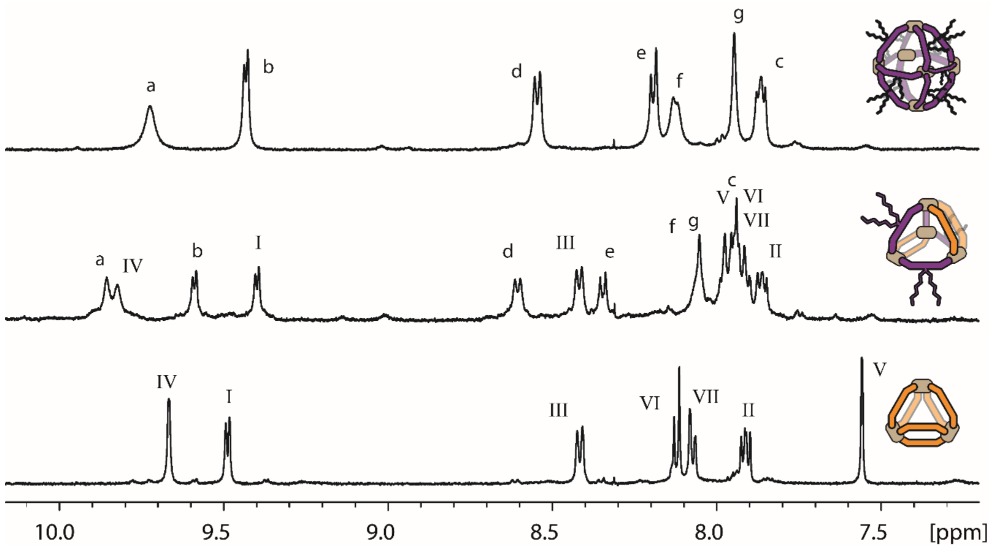

Figure S33: ^1H NMR spectrum (500 MHz, 298K, DMSO-d_6) of, top to bottom, $[Pd_6L^B{}_{12}]$, $[Pd_4L^A{}_4L^B{}_4]$ obtained mixing the two homoleptic assemblies, $[Pd_3L^A{}_6]$.

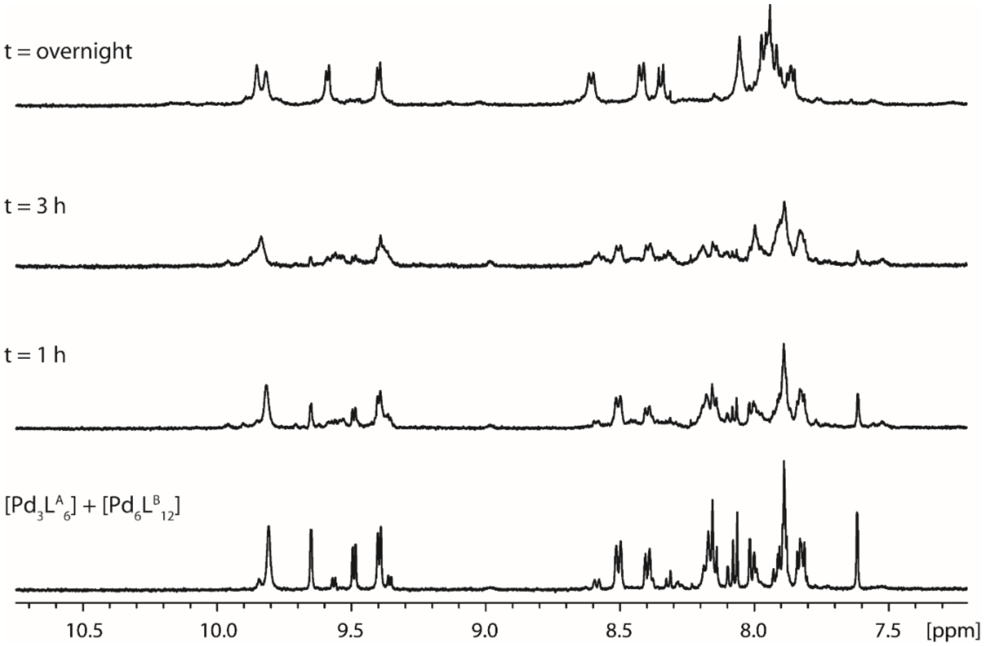

Figure S34: ^{1}H NMR spectra (500 MHz, 343K, DMSO-d_6) of the cage-to-cage transformation process followed over time. From bottom to top, fresh mixture of $[Pd_3L^A{}_6]$ and $[Pd_6L^B{}_{12}]$, reaction mixture after 1 h, 3 h and overnight at 70°C, respectively, yielding exclusively $[Pd_4L^A{}_4L^B{}_4]$ (compare Fig. S33).

2.3 DOSY NMR comparison

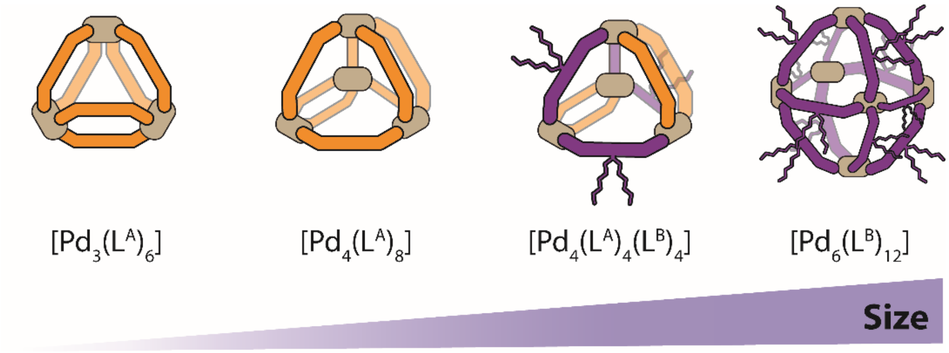

Scheme S3. Schemes of the reported assemblies

	$[Pd_3L^A{}_6]$	$[Pd_4L^A{}_8]$	$[Pd_4L^A{}_4L^B{}_4]$	$[Pd_6L^B{}_{12}]$
r_h in CD_3CN	11.04 Å	12.19 Å	--	15.99 Å
r_h in DMSO-d_6	13.20 Å	--	15.11 Å	18.66 Å

Table S1. Hydrodynamic radii in CD_3CN and DMSO-d_6 for all the reported species.

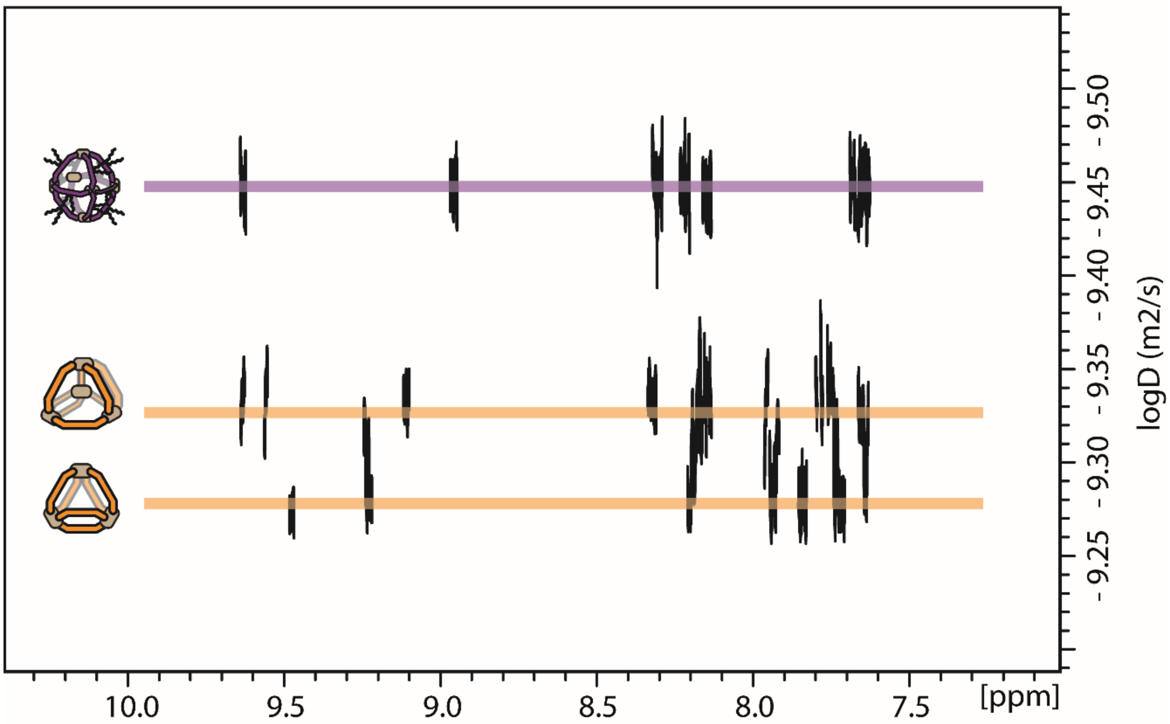

Figure S35. Overlap of ¹H DOSY spectra (500 MHz, 298K) for [$Pd_3L^A{}_6$], [$Pd_4L^A{}_8$] and [$Pd_6L^B{}_{12}$] in CD_3CN.

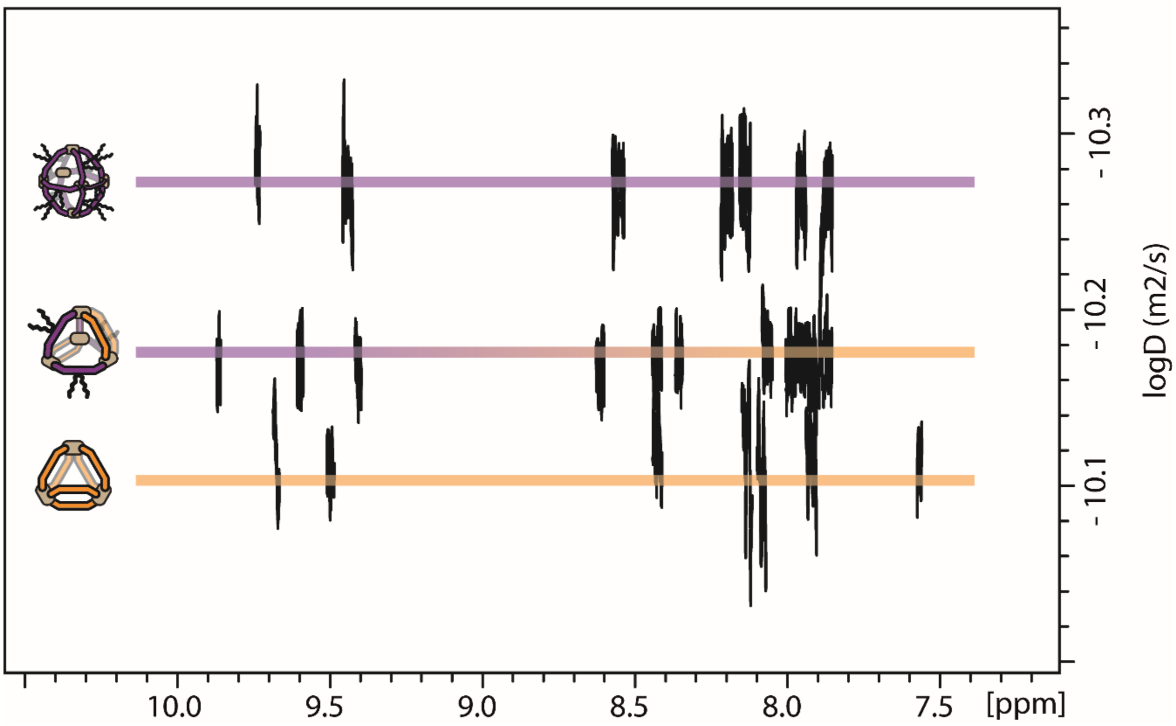

Figure S36. Overlap of ¹H DOSY spectra (500 MHz, 298K) for [$Pd_3L^A{}_6$], [$Pd_4L^A{}_4L^B{}_4$] and [$Pd_6L^B{}_{12}$] in DMSO-d_6.

3. Ligand binding modes

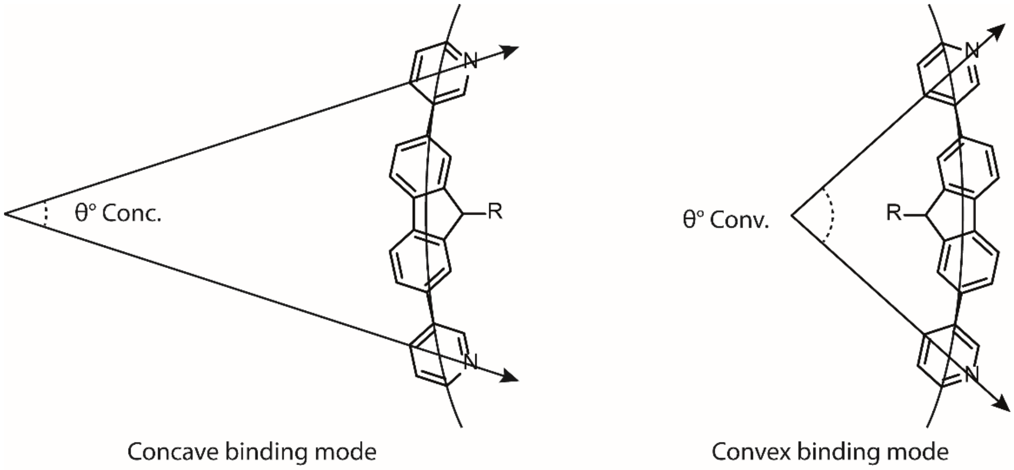

Scheme S4. Schematic representation of the concave and convex binding modes adopted by ligands L^A and L^B; θ° concave ≈ 40°, θ° convex ≈ 90°.

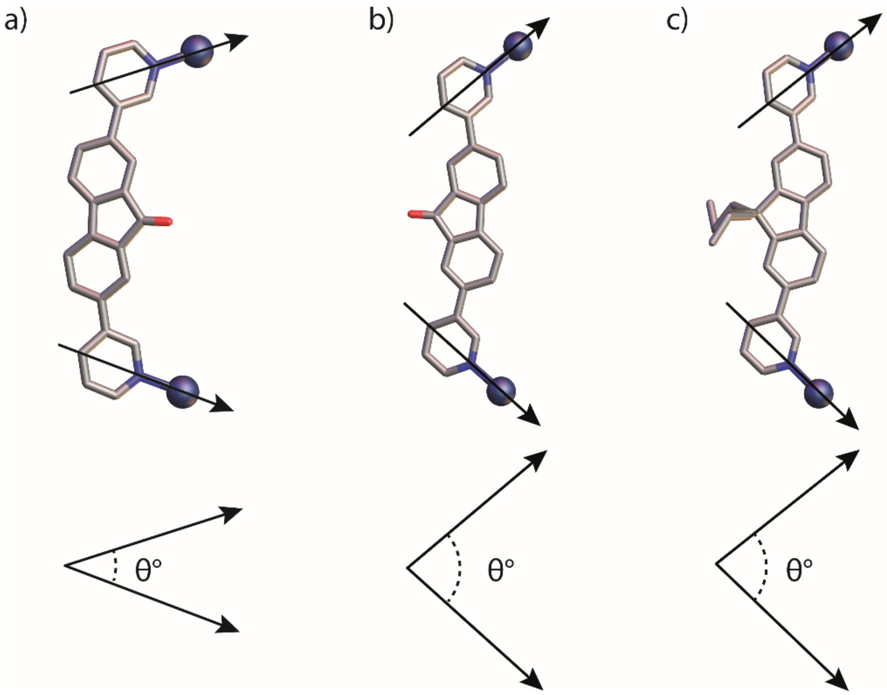

Figure S37. Comparison between the binding modes adopted in the crystal structures: a) concave binding mode in the homoleptic $[Pd_4L^A{}_8]$, θ° concave = 40.02°; b) convex binding mode in the homoleptic $[Pd_4L^A{}_8]$, θ° convex = 83.45°; c) convex binding mode for L^B in the heteroleptic $[Pd_4L^A{}_4L^B{}_4]$, θ° convex = 89.75°. The angles are measured between the Pd−N bonds.

Compound	Convex Pd-N to Pd-N angles θ°	Concave Pd-N to Pd-N angles θ°
$[Pd_3L^A_6]$		From 43.38° to 46.68°
$[Pd_4L^A_8]$	From 72.40° to 83.45°	From 39.58° to 43.70°
$[Pd_6L^A_{12}]$	From 68.29° to 71.65°	
$[Pd_6L^B_{12}]$	From 88.99° to 89.83°	
$[Pd_4L^A_4L^B_4]$	From 86.60° to 89.75°	From 34.37° to 37.59°

Table S2. Binding angles for $[Pd_4L^A_8]$, $[Pd_6L^A_{12}]$, $[Pd_6L^B_{12}]$ and $[Pd_4L^A_4L^B_4]$, measured as the angle between Pd−N bonds, for the same ligand.

4. Single crystal X-ray diffraction

4.1. Data collection details of $[Pd_3L^A_6]$ and $[Pd_6L^A_{12}]$

Suitable single crystals for X-ray structural analysis of $[Pd_3L^A_6]$ and $[Pd_6L^A_{12}]$ were mounted at room temperature in NVH oil. Crystals were stored in liquid nitrogen and measured at the beamline P11[S1] at Petra III synchrotron, DESY, Germany. UNI Pucks were transferred to the sample Dewar container and all samples were mounted using a StäubliTX60L robotic arm. A wavelength of 0.68880 Å was chosen using a liquid N_2 cooled double crystal monochromator. Single crystal X-ray diffraction data was collected at 80(2) K on a single axis goniometer, equipped with an Oxford Cryostream 800 and a Pilatus 6M detector. 3600 diffraction images were collected in a 360° ϕ sweep at a detector distance of 154.10 mm, 100% filter transmission, 0.1° step width and 100 milliseconds exposure time per image. Data integration and reduction were performed using XDS[S2] while the structural solution was obtained with SHELXT.[S3] The resolution was cut off at 0.70 Å for $[Pd_3L^A_6]$ and 0.84 Å for $[Pd_6L^A_{12}]$, after which the signal to noise ratio has dropped below $I/\sigma(I) < 2.0$. The structural models were refined against all data by full-matrix least-squares methods on F^2 using SHELXL2014[S4] in the framework of SHELXle.[S5] The SQUEEZE[S6] method provided by the program PLATON[S7] was used to improve the contrast of the electron density map the structure.

4.1.1. Refinement details for [$Pd_3L^A{}_6$] and [$Pd_6L^A{}_{12}$]

The anisotropic refinement for C, N, O atoms was enabled by a combination of similarity restraints (SIMU) and rigid bond restraints (RIGU). The contribution of the electron density from solvent molecules, which could not be modelled with discrete atomic positions were handled using the SQUEEZE routine in PLATON. The solvent mask file (.fab) computed by PLATON were included in the SHELXL refinement via the ABIN instruction leaving the measured intensities untouched.

4.2. Data collection details of [$Pd_4L^A{}_8$], [$Pd_6L^B{}_{12}$], [$Pd_4L^A{}_4L^B{}_4$] and L^B

Suitable single crystals for X–ray structural analysis of [$Pd_4L^A{}_8$], [$Pd_6L^B{}_{12}$], [$Pd_4L^A{}_4L^B{}_4$] and L^B were mounted at room temperature in NVH oil. Data was collected in-house on a Bruker D8 venture diffractometer equipped with an INCOATEC micro focus sealed tube (Iµs 3.0) using CuK_α radiation at 100 K. The resolution was cut off at 0.9 Å for [$Pd_4L^A{}_8$], 0.8 Å for [$Pd_6L^B{}_{12}$], 0.77 Å for [$Pd_4L^A{}_4L^B{}_4$] and L^B, after which the signal to noise ratio has dropped below $I/\sigma(I) < 2.0$. The data was integrated with APEX3. The structures were solved by intrinsic phasing/direct methods using SHELXT and refined with SHELXL for full-matrix least-squares routines on F^2 and ShelXle as a graphical user interface and the DSR program plugin was employed for modelling. For L^B, Olex2[S8] was used as a graphical user interface.

4.2.1. Refinement details for [$Pd_4L^A{}_8$], [$Pd_6L^B{}_{12}$], [$Pd_4L^A{}_4L^B{}_4$] and L^B

Stereochemical restraints for organic ligands in residues FCO and JLE were generated by the GRADE program using the GRADE Web Server (http://grade.globalphasing.org) and applied in the refinement. The GRADE dictionary contains target values and standard deviations for 1,2-distances (DFIX) and 1,3-distances (DANG), as well as restraints for planar groups (FLAT). The anisotropic refinement for C, N, O atoms was enabled by a combination of similarity restraints (SIMU) and rigid bond restraints (RIGU).[12] The contribution of the electron density from solvent molecules, which could not be modelled with discrete atomic positions were handled using the SQUEEZE routine in PLATON. The solvent mask file (.fab) computed by PLATON were included in the SHELXL refinement via the ABIN instruction leaving the measured intensities untouched.

Table S3. Crystallographic data of [$Pd_3L^A{}_6$], [$Pd_6L^A{}_{12}$], [$Pd_4L^A{}_8$], [$Pd_6L^B{}_{12}$], [$Pd_4L^A{}_4L^B{}_4$] and L^B

Compound	[$Pd_3L^A{}_6$]	[$Pd_6L^A{}_{12}$]	L^B
CCDC number	2061340	2061345	2061351
Identification code	jt30c_9_sq	jt30d_sq	JT63_0m2
Empirical formula	C154 H132 B6 F24 N12 O14 Pd3 S8	C336 H288 B12 F48 N24 O42 Pd6	C35 H40 N2
Formula weight	3471.25	7014.01	488.69
Temperature (K)	80(2) K	80(2) K	200(2) K
Wavelength (Å)	0.6888 Å	0.6888	1.54178 Å
Crystal system	Triclinic	Trigonal	Triclinic
Space group	*P*-1	*R*-3	*P*-1
a (Å)	19.658(4)	46.225(7)	12.0797(2)
b (Å)	20.769(4)	46.225(7)	16.1115(2)
c (Å)	25.381(5)	46.312(9)	17.3409(3)
α (°)	113.12(3)	90	111.9060(10)
β (°)	100.63(3)	90	92.0430(10)
γ (°)	95.19(3)	120	108.8500(10)
Volume (Å^3)	9213(4)	85699(30)	2916.04(8)
Z	2	6	4
Density (calc.) (Mg/m^3)	1.251	0.815	1.113
Absorption coefficient (mm^{-1})	0.417	0.220	0.483
F (000)	3528	21456	1056
Crystal size (mm^3)	0.310 x 0.030 x 0.020	0.240 x 0.040 x 0.030	0.300 x 0.300 x 0.100
θ range for data collection (°)	0.871 to 29.474	0.652 to 24.204	2.791 to 65.083°.
Index ranges	-27<=h<=28, -29<=k<=29, -36<=l<=36	-54<=h<=55, -55<=k<=55, -51<=l<=53	-14<=h<=14, -18<=k<=18, -20<=l<=20
Reflections collected	155854	163982	182065
Independent reflections [R(int)]	48090 [R(int) = 0.0676]	33256 [R(int) = 0.0654]	9933 [R(int) = 0.2792]
Completeness to θ	91.50%	98.80%	99.80%
Absorption correction	None	None	Semi-empirical from equivalents
Refinement method	Full-matrix least-squares on F^2	Full-matrix least-squares on F^2	Full-matrix least-squares on F^2
Data / restraints / parameters	48090 / 873 / 2062	33256 / 1280 / 1571	9933 / 0 / 671
Goodness-of-fit on F^2	1.759	1.035	1.046
Final R indices [I>2σ(I)]	R1 = 0.0799, wR2 = 0.2498	R1 = 0.0768, wR2 = 0.2494	R1 = 0.0949, wR2 = 0.2564
R indices (all data)	R1 = 0.0855, wR2 = 0.2560	R1 = 0.0958, wR2 = 0.2656	R1 = 0.1033, wR2 = 0.2711

Table S3. *(Continued)*

Compound	$[Pd_4L^A{}_8]$	$[Pd_6L^B{}_{12}]$	$[Pd_4L^A{}_4L^B{}_4]$
CCDC number	2061344	2061347	2061350
Identification code	cu_jt31c_0m_sq	jt69_pd6le12_oh_sq	jt70a_sq
Empirical formula	C206 H162 B8 F32 N22 O16 Pd4	C432 H516 B8 F32 N24 O6 Pd6 S6	C250 H270 B6 F24 N16 O13 Pd4 S9
Formula weight	4321.65	7665.9	4941.8
Temperature (K)	100(2) K	100(2) K	100(2) K
Wavelength (Å)	1.54178 Å	1.54178 Å	1.54178 Å
Crystal system	Triclinic	Trigonal	Monoclinic
Space group	*P*-1	*P*-31*c*	*C*2
a (Å)	25.4006(16)	26.1811(9)	51.861(3)
b (Å)	26.4173(18)	26.1811(9)	29.8575(15)
c (Å)	27.0995(17)	49.960(3)	21.7468(11)
α (°)	114.582(3)	90	90
β (°)	92.225(3)	90	98.549(3)
γ (°)	115.550(3)	120	90
Volume ($Å^3$)	14381.5(17)	29657(3)	33299(3)
Z	2	2	4
Density (calc.) (Mg/m^3)	0.998	0.858	0.986
Absorption coefficient (mm^{-1})	2.564	2.040	2.728
F (000)	4384	8048	10240
Crystal size (mm^3)	0.300 x 0.040 x 0.040	0.120 x 0.120 x 0.120	0.200 x 0.200 x 0.100
θ range for data collection (°)	2.000 to 59.338	1.768 to 47.234	1.712 to 59.832
Index ranges	-28<=h<=28, -29<=k<=29, -30<=l<=28	-24<=h<=24, -24<=k<=24, -47<=l<=47	-57<=h<=57, -33<=k<=33, -24<=l<=24
Reflections collected	79870	556958	182849
Independent reflections [R(int)]	37113 [R(int) = 0.0363]	8967 [R(int) = 0.1665]	47487 [R(int) = 0.0777]
Completeness to θ	88.70%	100.00%	98.10%
Absorption correction	Semi-empirical from equivalents	Semi-empirical from equivalents	Semi-empirical from equivalents
Max. and min. transmission	0.904 and 0.513	0.792 and 0.792	0.772 and 0.611
Refinement method	Full-matrix least-squares on F^2	Full-matrix least-squares on F^2	Full-matrix least-squares on F^2
Data / restraints / parameters	37113 / 5168 / 2641	8967 / 1656 / 828	47487 / 6903 / 3276
Goodness-of-fit on F^2	1.078	1.873	1.041
Final R indices [I>2σ(I)]	R1 = 0.0840, wR2 = 0.2381	R1 = 0.1242, wR2 = 0.3940	R1 = 0.0798, wR2 = 0.2019
R indices (all data)	R1 = 0.1040, wR2 = 0.2690	R1 = 0.1504, wR2 = 0.4269	R1 = 0.1261, wR2 = 0.2440
Largest diff. peak and hole ($e.Å^{-3}$)	n/a	n/a	n/a
Absolute structure parameter			0.491(5)

5. Photophysical studies

UV vis spectra were recorded on a DAD HP-8453 UV-Vis spectrometer.

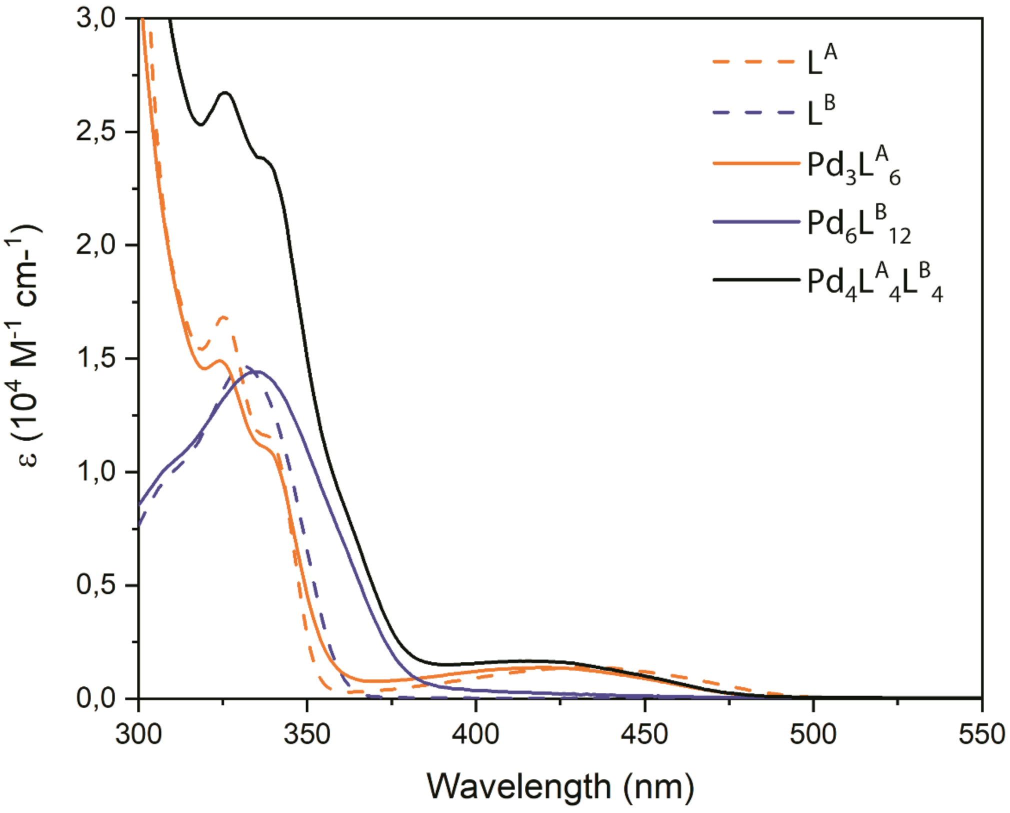

Figure S38. UV-Vis absorption spectra of L^A, L^B, [$Pd_3L^A_6$], [$Pd_4L^A_4L^B_4$] and [$Pd_6L^B_{12}$] in DMSO, concentration $5.0*10^{-4}$ M (referring to ligand concentration); cuvette path length: 0.1 cm.

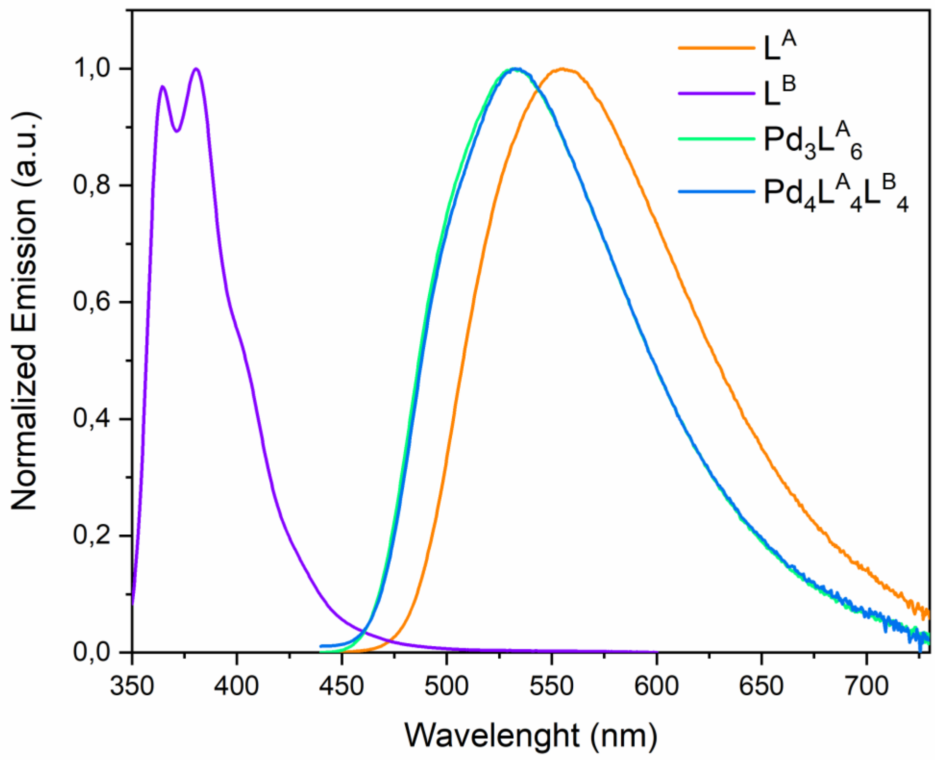

Figure S39. Normalized emission spectra of L^A, [$Pd_3L^A_6$] and [$Pd_4L^A_4L^B_4$] in DMSO, 1,4*10^{-4} M, referring to ligand concentration, λ_{ex} = 430 nm; L^B 1,4*10^{-4} M, λ_{ex} = 330 nm. [$Pd_6L^B_{12}$] does not show emission.

6. Host-Guest studies

Host-guest studies have been performed by ^{1}H NMR titrations and ESI-MS analysis on a series of three bis-sulfonate guest molecules G1, G2 and G3, as *tert*-butylammonium salts. Due to signal broadening or even precipitation after adding 1 equivalent of guest molecule, it was not possible to determine association constants. However, clear signal shifting is observed in all cases, involving mainly the inward pointing protons, H_{IV} and H_V for [$Pd_3L^A_6$], H_a, H_e and H_f for [$Pd_6L^B_{12}$], and H_{IV}, H_a, H_e and H_f for [$Pd_4L^A_4L^B_4$], as expected for guests binding inside the cage cavity.

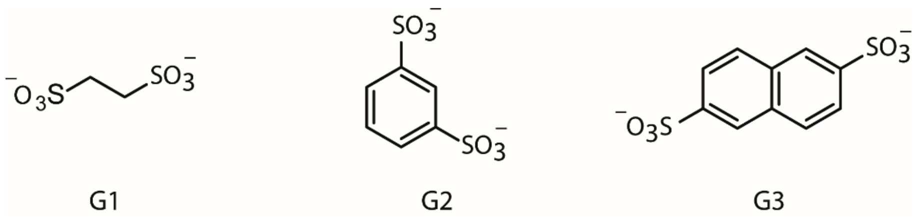

Scheme S5. List of guest molecules.

6.1. Host-guest studies of [$Pd_3L^A_6$].

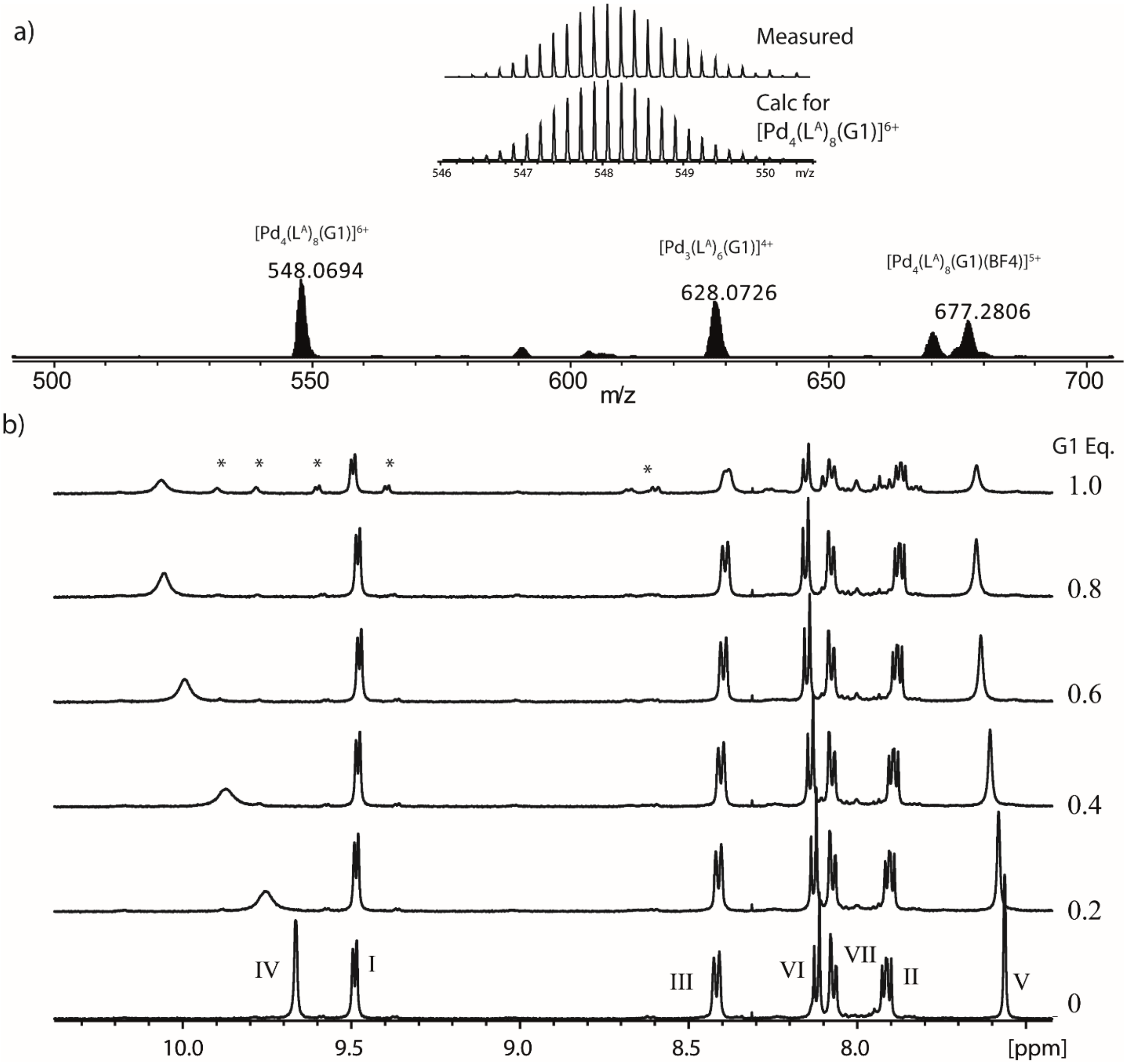

Figure S40. Host-guest interaction of [$Pd_3L^A_6$] with G1 in DMSO-d$_6$; a) ESI-MS shows interaction of G1 with [$Pd_3L^A_6$] and [$Pd_4L^A_8$], isotopic pattern for [$Pd_4L^A_8$(G1)]$^{6+}$ shown in the inset; b) ^{1}H NMR titration spectra (500 MHz, 298K, DMSO-*d$_6$*) of [$Pd_3L^A_6$] upon addition of G1. The spectrum after addition of 1 equivalent G1 has been recorded after 2 hours, showing appearance of signals ascribed to [$Pd_4L^A_8$], indicated by *.

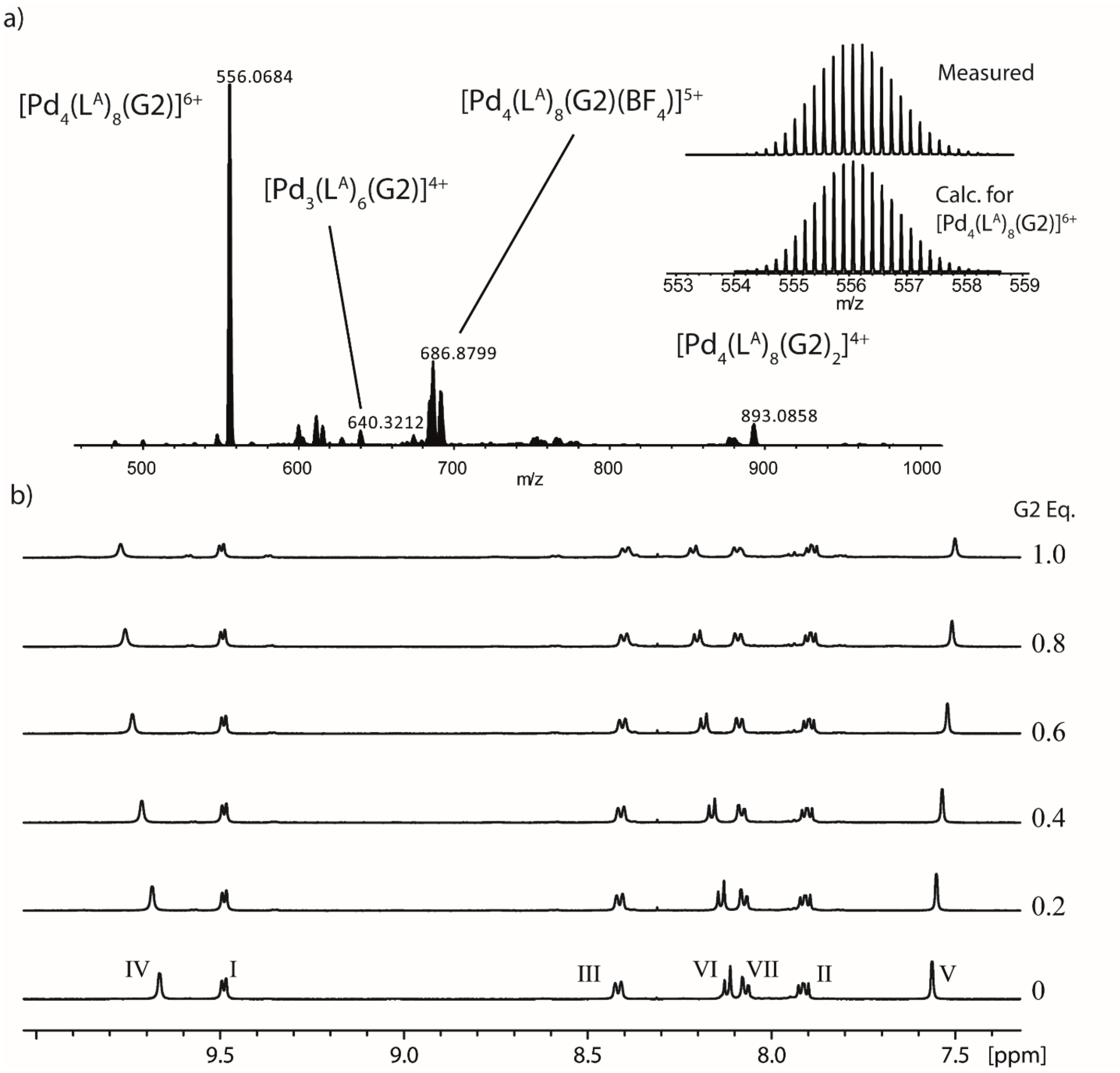

Figure S41. Host-guest interaction of $[Pd_3L^A{}_6]$ with G2 in DMSO-d_6; a) ESI-MS shows interaction of one G2 with $[Pd_3L^A{}_6]$ and up to two G2 with $[Pd_4L^A{}_8]$, isotopic pattern for $[Pd_4L^A{}_8(G2)]^{6+}$ shown in the inset; b) ^1H NMR titration spectra (500 MHz, 298K, DMSO-d_6) of $[Pd_3L^A{}_6]$ upon addition of G2.

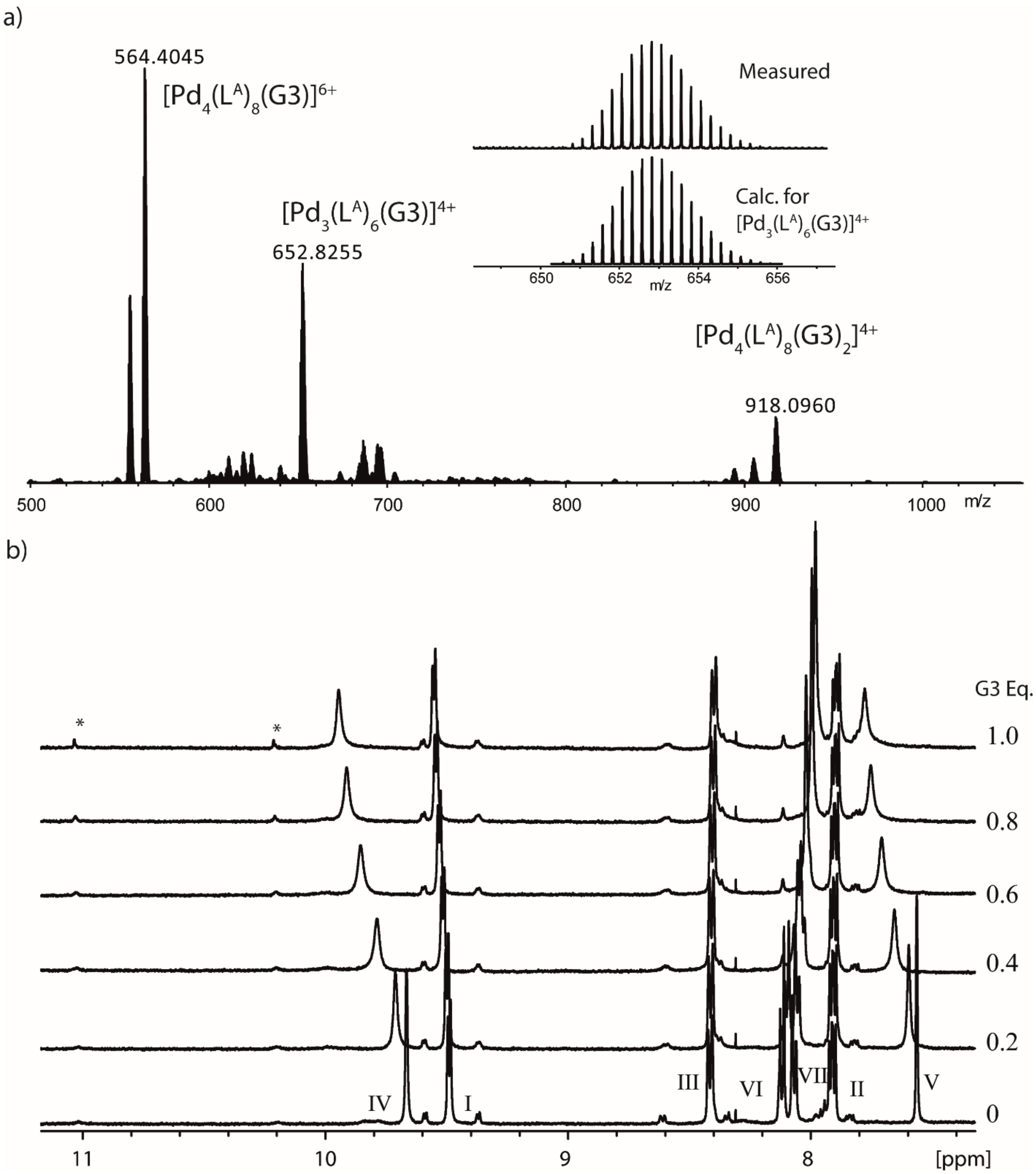

Figure S42. Host-guest interaction of $[Pd_3L^A{}_6]$ with G3 in DMSO-d$_6$; a) ESI-MS shows interaction of one G3 with $[Pd_3L^A{}_6]$ and up to two G3 with $[Pd_4L^A{}_8]$, isotopic pattern for $[Pd_3L^A{}_6(G3)]^{4+}$ shown in the inset; b) ^{1}H NMR titration spectra (500 MHz, 298K, DMSO-d_6) of $[Pd_3L^A{}_6]$ upon addition of G3. * indicates guest signals.

6.2. Host-guest studies of $[Pd_6L^B{}_{12}]$.

a)

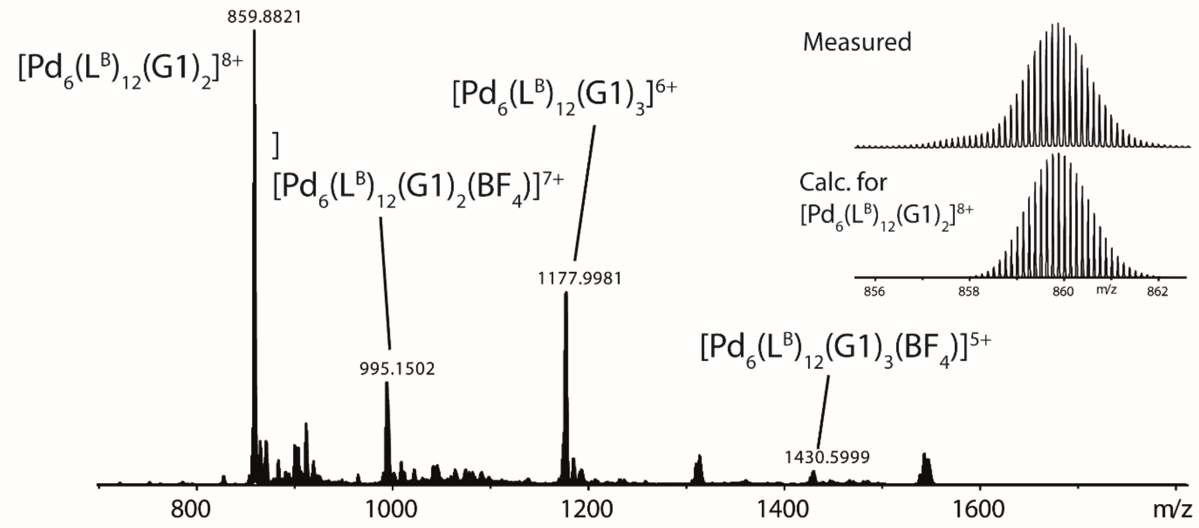

b)

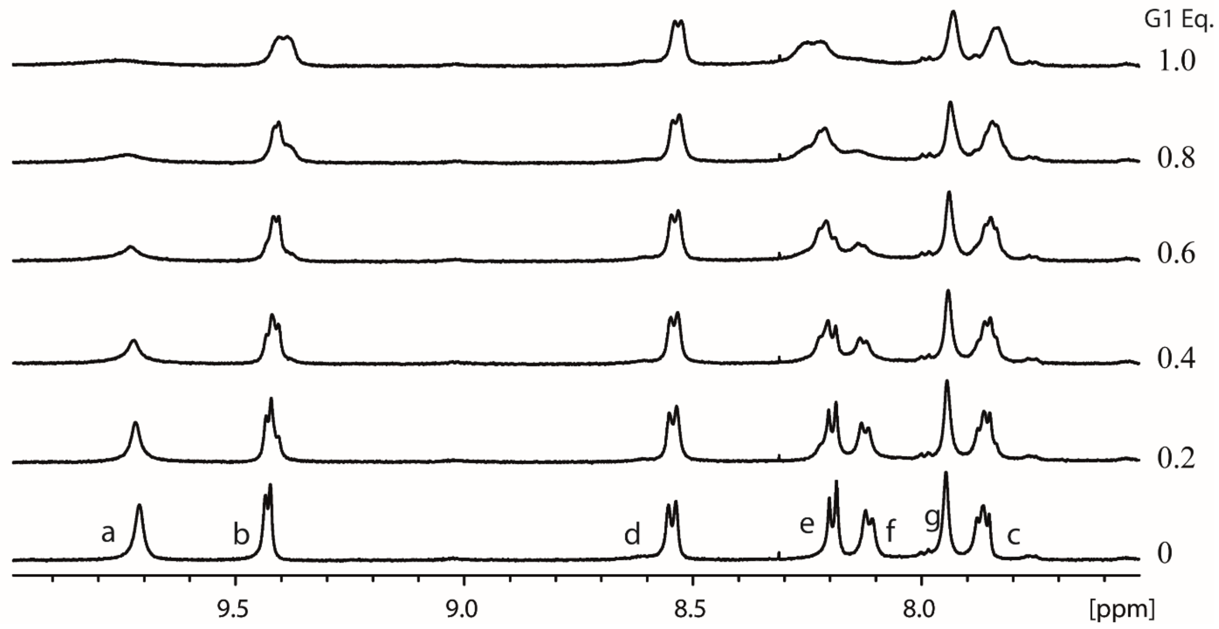

Figure S43. Host-guest interaction of $[Pd_6L^B{}_{12}]$ with G1 in DMSO-d_6; a) ESI-MS shows interaction of up to three G1 with $[Pd_6L^B{}_{12}]$, isotopic pattern for $[Pd_6L^B{}_{12}(G1)_2]^{8+}$ shown in the inset; b) ^1H NMR titration spectra (500 MHz, 298K, DMSO-d_6) of $[Pd_6L^B{}_{12}]$ upon addition of G1.

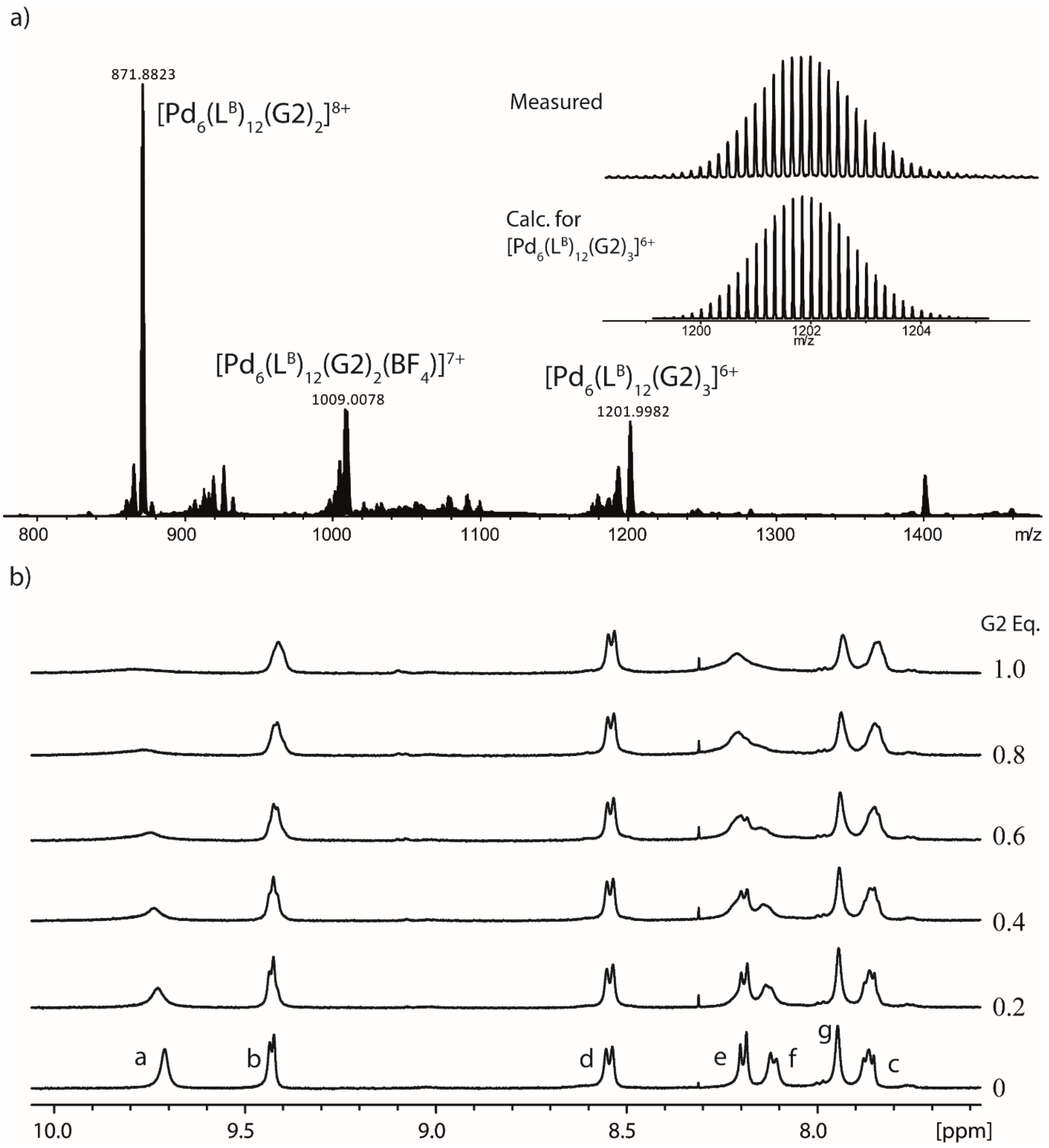

Figure S44. Host-guest interaction of $[Pd_6L^B{}_{12}]$ with G2 in DMSO-d_6; a) ESI-MS shows interaction of up to three G2 with $[Pd_6L^B{}_{12}]$, isotopic pattern for $[Pd_6L^B{}_{12}(G2)_3]^{6+}$ shown in the inset; b) ^1H NMR titration spectra (500 MHz, 298K, DMSO-d_6) of $[Pd_6L^B{}_{12}]$ upon addition of G2.

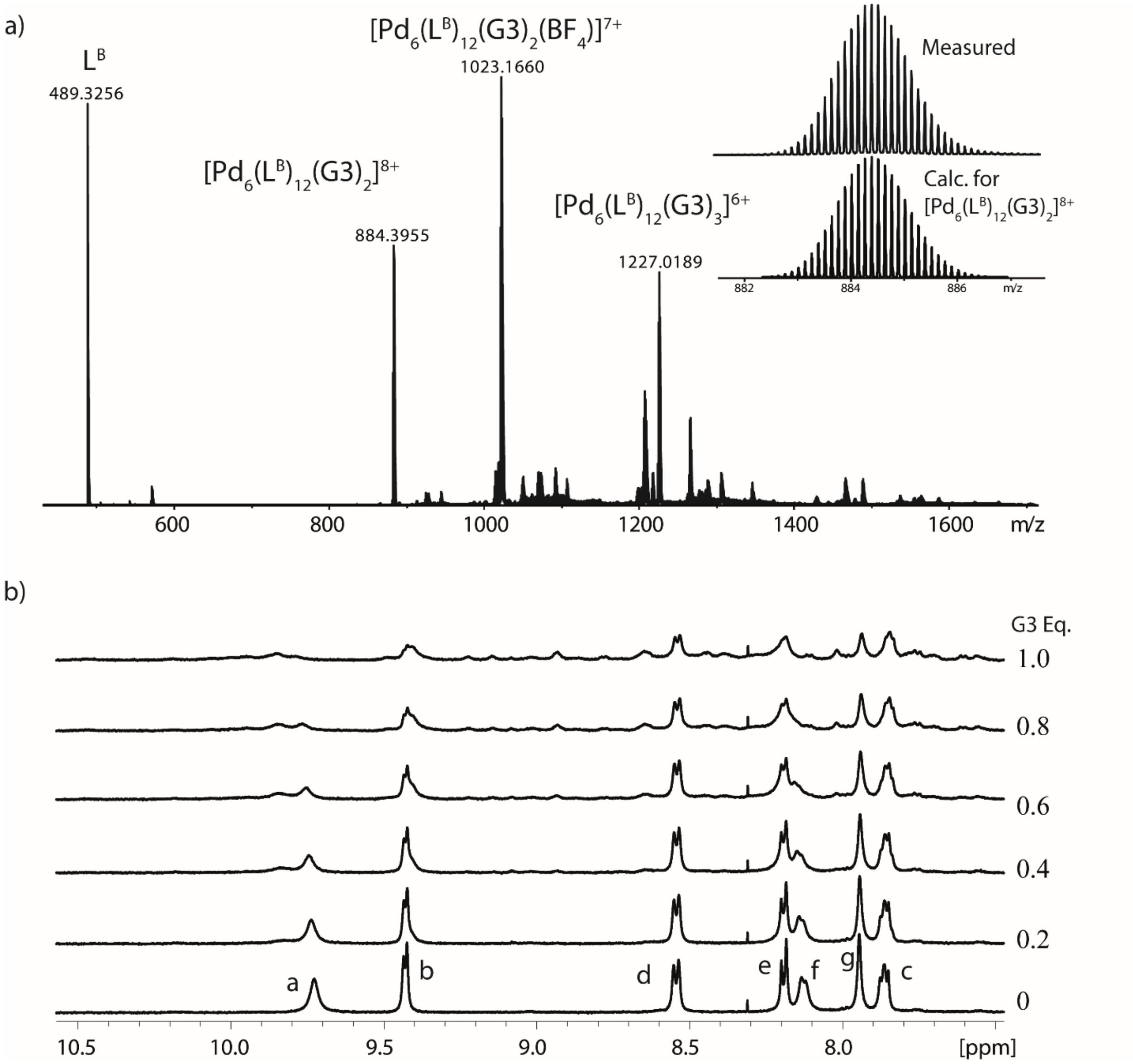

Figure S45. Host-guest interaction of $[Pd_6L^B{}_{12}]$ with G3 in DMSO-d$_6$; a) ESI-MS shows interaction of up to three G3 with $[Pd_6L^B{}_{12}]$, isotopic pattern for $[Pd_6L^B{}_{12}(G3)_2]^{8+}$ shown in the inset; b) ^{1}H NMR titration spectra (500 MHz, 298K, DMSO-d_6) of $[Pd_6L^B{}_{12}]$ upon addition of G3.

6.3. Host-guest studies of [$Pd_4L^A{}_4L^B{}_4$].

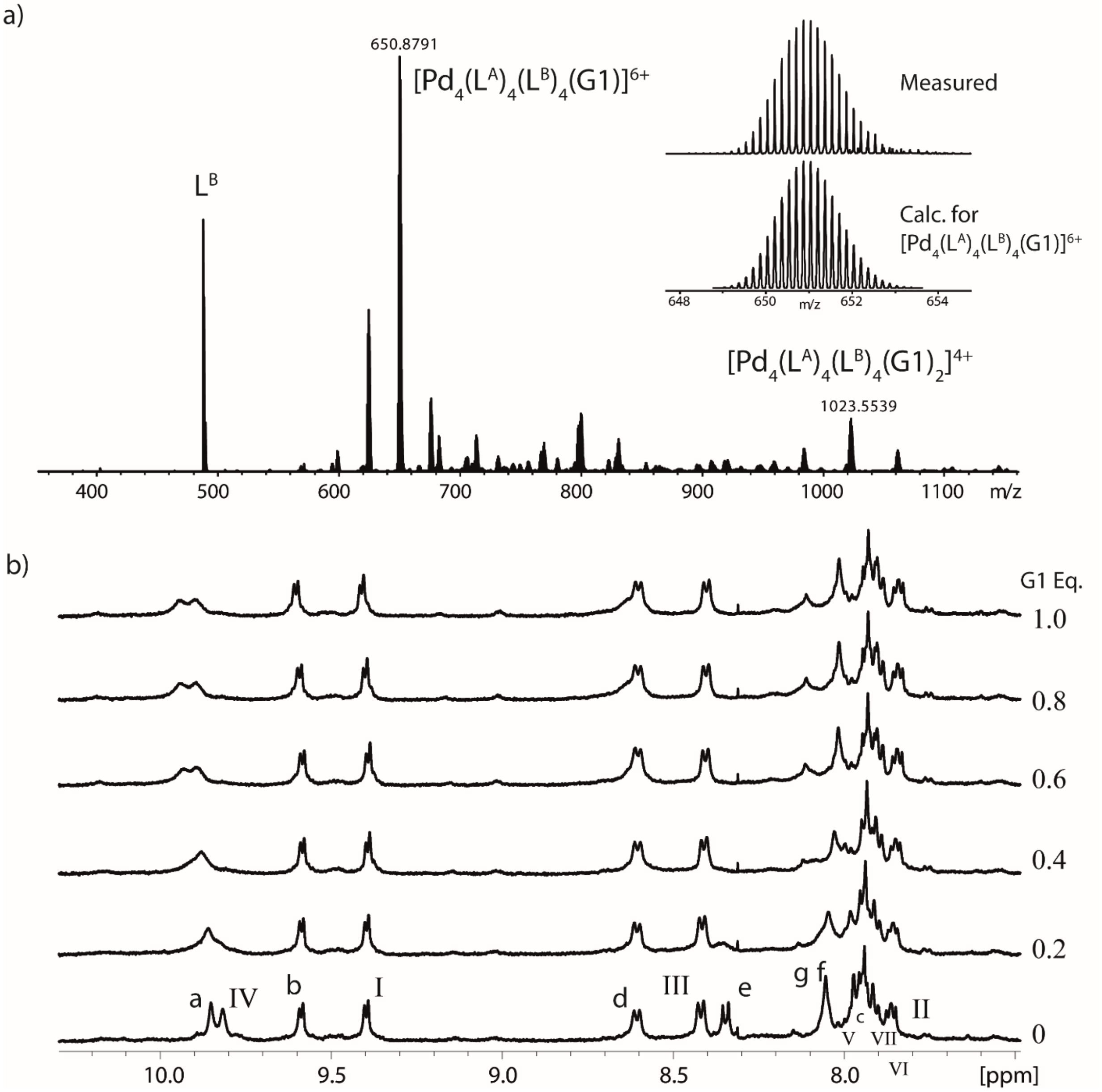

Figure S46. Host-guest interaction of [$Pd_4L^A{}_4L^B{}_4$] with G1 in DMSO-d$_6$; a) ESI-MS shows interaction of up to two G1 with [$Pd_4L^A{}_4L^B{}_4$], isotopic pattern for [$Pd_4L^A{}_4L^B{}_4$(G1)]$^{6+}$ shown in the inset; b) ^{1}H NMR titration spectra (500 MHz, 298K, DMSO-*d$_6$*) of [$Pd_4L^A{}_4L^B{}_4$] upon addition of G1.

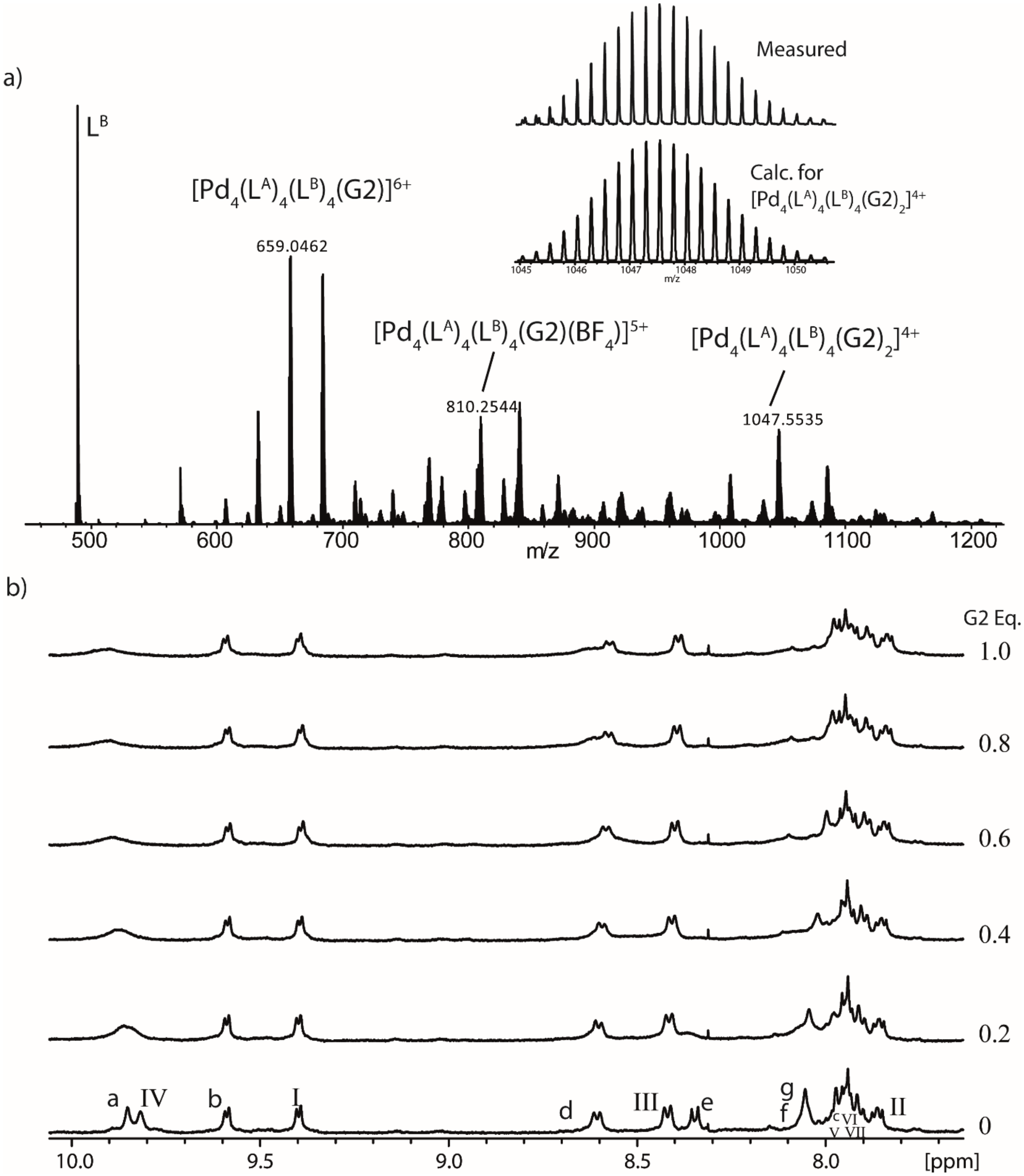

Figure S47. Host-guest interaction of $[Pd_4L^A_4L^B_4]$ with G2 in DMSO-d_6; a) ESI-MS shows interaction of up to two G2 with $[Pd_4L^A_4L^B_4]$, isotopic pattern for $[Pd_4L^A_4L^B_4(G2)_2]^{4+}$ shown in the inset; b) ^1H NMR titration spectra (500 MHz, 298K, DMSO-*d*$_6$) of $[Pd_4L^A_4L^B_4]$ upon addition of G2.

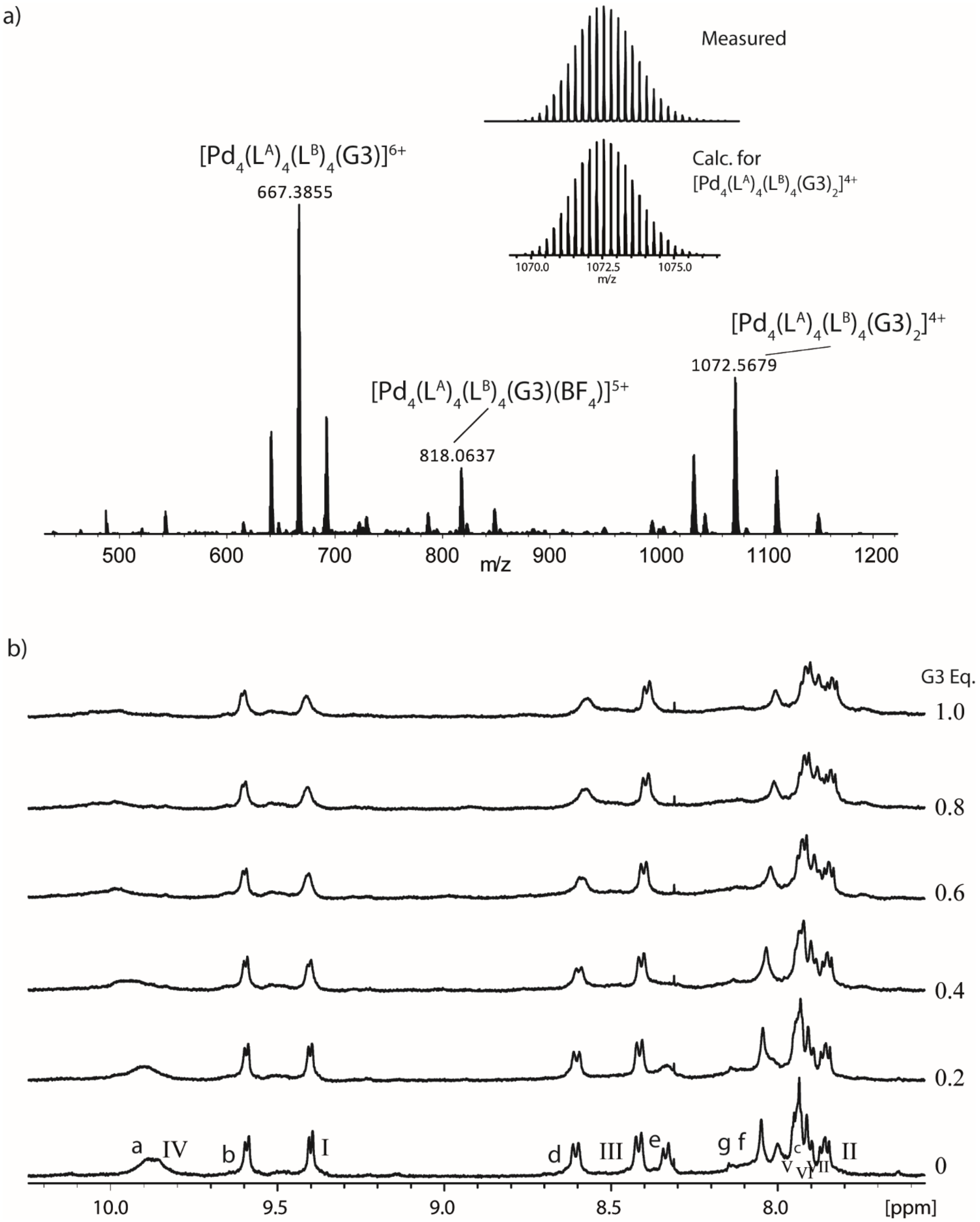

Figure S48. Host-guest interaction of $[Pd_4L^A_4L^B_4]$ with G3 in DMSO-d$_6$; a) ESI-MS shows interaction of up to two G3 with $[Pd_4L^A_4L^B_4]$, isotopic pattern for $[Pd_4L^A_4L^B_4(G3)_2]^{4+}$ shown in the inset; b) ^{1}H NMR titration spectra (500 MHz, 298K, DMSO-*d$_6$*) of $[Pd_4L^A_4L^B_4]$ upon addition of G3.

7. References

[S1] Burkhardt, A.; Pakendorf, T.; Reime, B.; Meyer, J.; Fischer, P.; Stübe, N.; Panneerselvam, S.; Lorbeer, O.; Stachnik, K.; Warmer, M.; Rödig, P.; Göries, D. and Meents, A. Eur. Phys. J. Plus., **2016**, *131*, 56.

[S2] Kabsch, W. Acta Crystallogr. Sect. D, **2010**, *66*, 125.

[S3] Sheldrick, G. M. Acta Crystallogr. Sect. A, **2015**, *71*, 3.

[S4] Sheldrick, G. M. Acta Crystallogr. Sect. A, **2008**, *64*, 112.

[S5] Hubschle, C. B.; Sheldrick, G. M. and Dittrich, B. J. Appl. Crystallogr., **2011**, *44*, 1281.

[S6] Spek,A. Acta Crystallogr. Sect. C, **2015**, *71*, 9.

[S7] Spek,A. Acta Crystallogr. Sect. D, **2009**, *65*, 148.

[S8] Dolomanov, O.V., Bourhis, L.J., Gildea, R.J, Howard, J.A.K. & Puschmann, H., *J. Appl. Cryst.*, **2009**, *42*, 339.
